# Characterizing the tumor suppressor activity of FLCN in Birt-Hogg-Dubé syndrome cell models through transcriptomic and proteomic analysis

**DOI:** 10.1038/s41388-025-03325-z

**Published:** 2025-03-25

**Authors:** Rachel-Ann Jones, Elaine A. Dunlop, Jesse D. Champion, Peter F. Doubleday, Tijs Claessens, Zahra Jalali, Sara Seifan, Iain A. Perry, Peter Giles, Oliver Harrison, Barry J. Coull, Arjan C. Houweling, Arnim Pause, Bryan A. Ballif, Andrew R. Tee

**Affiliations:** 1 Division of Cancer and Genetics, Cardiff University, Heath Park, Cardiff, CF14 4XN UK; 2 Department of Biology, University of Vermont, Marsh Life Science 311, 109 Carrigan Drive, Burlington, VT USA; 3 Department of Biochemistry and Goodman Cancer Institute, McGill University, Montréal, QC Canada; 4 Lancaster Medical School, Lancaster University, Lancaster, LA1 4AT UK; 5 Department of Human Genetics, Amsterdam UMC, Vrije Universiteit Amsterdam, Amsterdam, The Netherlands

**Keywords:** Cancer genetics, Mechanisms of disease, Transcription, Proteomics

## Abstract

Birt-Hogg-Dubé syndrome (BHD) patients are uniquely susceptible to all renal tumor subtypes. However, the underlying mechanism of carcinogenesis is unclear. To study cancer development in BHD, we used human proximal kidney (HK2) cells and found that long-term folliculin (*FLCN*) knockdown was required to increase the tumorigenic potential of these cells, as evidenced by the formation of larger spheroids under nonadherent conditions. Transcriptomic and proteomic analyses revealed links between the FLCN, cell cycle control and DNA damage response (DDR) machinery. In addition, HK2 cells lacking *FLCN* had an altered transcriptome profile and enriched cell cycle control genes. G_1_/S cell cycle checkpoint signaling was compromised by increased protein levels of cyclin D1 (CCND1) and hyperphosphorylation of retinoblastoma 1 (RB1). A FLCN interactome screen revealed that FLCN binds to DNA-dependent protein kinase (DNA-PK). This novel interaction was reversed in an irradiation-responsive manner. Knockdown of *FLCN* in HK2 cells caused a marked increase in γH2AX and RB1 phosphorylation. The levels of both CCND1 and phosphorylated RB1 remained high during DNA damage, which was associated with defective cell cycle control caused by *FLCN* knockdown. Furthermore, *Flcn*-knockdown *C. elegans* were defective in cell cycle arrest caused by DNA damage. This work revealed that long-term *FLCN* loss and associated cell cycle defects in BHD patients could contribute to their increased risk of cancer.

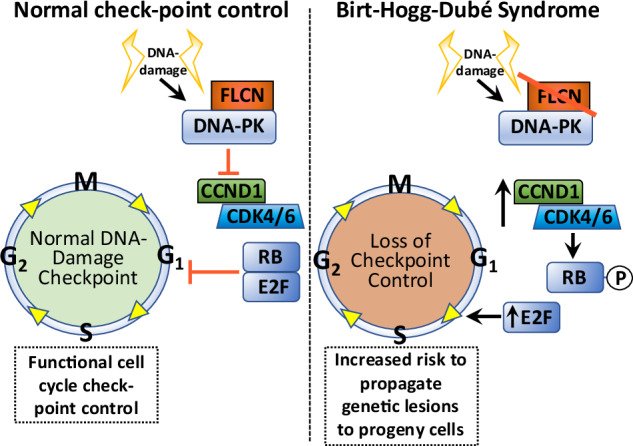

## Introduction

Birt-Hogg-Dubé syndrome (BHD, OMIM # 135150) is an autosomal dominant condition clinically characterized by fibrofolliculomas, pneumothorax associated with multiple lung cysts and early-onset multiple renal cell cancers (RCC) [1, 2]. BHD is caused by inactivating germline variants in the *FLCN* (folliculin) gene [2]. The lifetime risk of RCC in patients with BHD has been estimated to be approximately 15%. BHD patient renal tumors may present as a variety of histological subtypes, including mixed forms [3]. Most BHD-related renal cell cancers grow slowly, but metastatic disease may present as the first manifestation of the disease. Surveillance for early detection of renal lesions and surgical treatment of localized RCC are based on insight into the biological behavior of these tumors. Many BHD patients develop multiple bilateral renal tumors, and nephron-sparing surgery is therefore essential [2]. There is evidence of biallelic inactivation of *FLCN* in many BHD RCCs. Targeted treatments for such tumors are not currently available due to a lack of basic understanding of the key pathways involved in tumor growth.

Although major advances have been made, the underlying mechanism of how BHD tumors develop over time has not been determined. Current evidence indicates that the core function of FLCN is in lysosomal nutrient sensing and autophagy control through FLCN’s control of the Rag GTPases and thus mechanistic target of rapamycin (mTOR) phosphorylation of TFE3 and TFEB [4–7]. Further crosstalk with lysosomal pathways likely occurs via both AMPK- and ULK1-mediated phosphorylation of FLCN [8] and TFEB/TFE3 phosphorylation via AMPK [9]. As a direct or indirect result of lysosomal dysfunction, loss of *FLCN* can cause abnormal ciliogenesis [10] and defects in metabolic homeostasis [11, 12], with the metabolic shift seen in BHD cells resulting from FLCN inactivation or dissociation from LDHA [13]. FLCN has also been linked to a variety of fundamental cellular processes, including cell adhesion through plakophilin-4 (PKP4) [14, 15], cell growth and division through rRNA synthesis [16], cyclin D1 (CCND1) and marked changes in gene expression [17]. RagC-independent functions of FLCN have also been revealed in recent research, where FLCN was found to be a Rab7A GTPase-activating protein involved in endocytic trafficking of epidermal growth factor receptor [18]. A related novel hereditary disorder was linked to *PR/SET domain 10* (*PRDM10*), which predisposes families to skin and mucosal lesions, lipomatosis and renal cell carcinomas [19, 20]. The *PRDM10* mutation that cosegregated with disease was found to be defective at binding to *FLCN* promoter regions and consequently caused a reduction in FLCN expression [19].

Although the involvement of FLCN in cell growth control is better understood, it is still not clear how dysregulation of this pathway following FLCN loss could predispose patients to multiple RCC subtypes with different mutational signatures [21]. Therefore, we wanted to further study the tumorigenic mechanisms underlying *FLCN* loss. To do this, we studied a human kidney proximal tubule (HK2) cell line, as proximal tubule cells in the kidney are considered to be the origin cell in BHD tumor formation [22–24]. We observed increased tumorigenic properties after long-term knockdown of *FLCN* in HK2 cells (after they had been cultured for one year), while short-term *FLCN* knockdown had little ability to transform these cells. We examined the transcriptome profiles of these cells to reveal the enrichment of genes linked to cell cycle control, a tumorigenic feature that became more prominent with long-term loss of *FLCN*. To better understand the function of FLCN, we then carried out proteomic analysis of FLCN interactors, which were also mapped to multiple cell cycle regulators and included components of the DNA damage machinery. Our findings suggest that alterations in cell cycle control upon *FLCN* loss enhance cancer progression in patients with BHD.

## Results

### Long-term knockdown of *FLCN* increases tumorigenesis

**Fig. 1:**
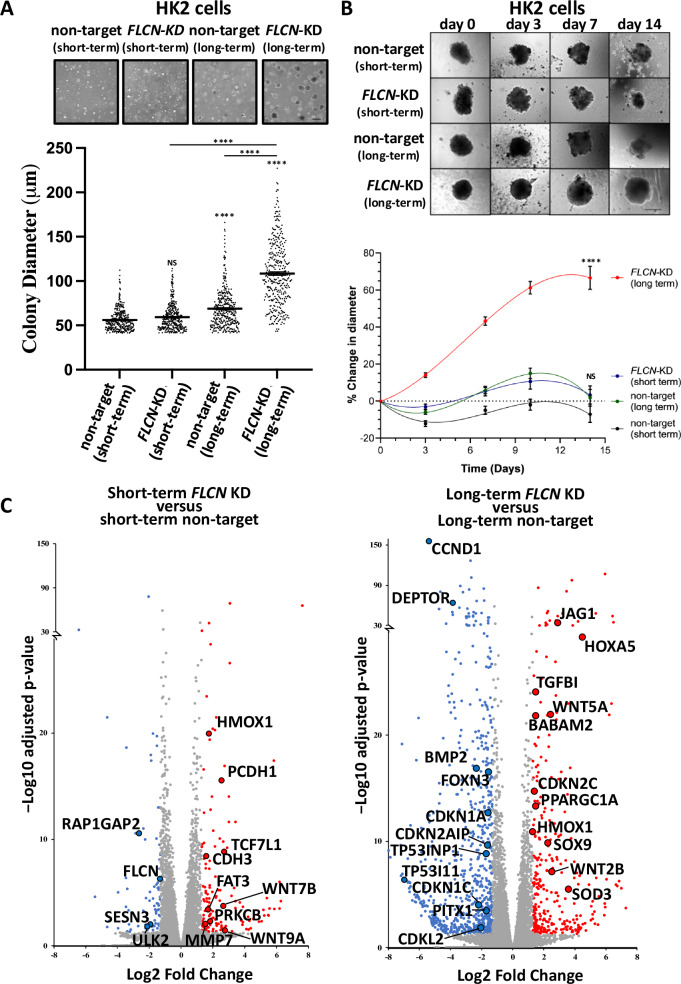
Long-term knockdown of *FLCN* increases tumorigenesis. **A** HK2 cells with or without short-term or long-term *FLCN* shRNA knockdown (compared to cells with nontargeting shRNA) were grown in soft agar for 21 days. The colony diameter was measured using ImageJ. (v1.53t), and a representative image of the tumors is shown (**s**calebar 250 μm), with the distribution of the tumors graphed. *n* = 360 per condition over 6 biological samples. Each data point represents a single tumor. Stats: data were not normally distributed (according to D’Agostino and Pearson tests) and were analyzed using nonparametric Kruskai-Wallis ANOVA with Dunn’s multiple comparisons test. **B** These HK2 cells were also plated under nonadherent conditions and imaged over 14 days (**s**calebar 250 μm), and the percentage change in diameter was plotted over time (*n* = 42). **C** RNA sequencing of HK2 cells with short- or long-term nontargeting or *FLCN* shRNA knockdown was carried out (*n* = 3). Differential gene expression was compared between short-term *FLCN* knockdown and nontarget shRNA knockdown and long-term *FLCN* knockdown and nontarget shRNA knockdown HK2 cells. Volcano plots are shown with the following thresholds: ≤ & ≥ 2.5-fold change and padj < 0.05 with false rate discovery (FDR) correction applied.

To mimic long-term haploinsufficiency of *FLCN* in kidney cells from BHD patients, we continuously grew HK2 cells, a human proximal tubule cell line, with or without *FLCN* knockdown for one year in tissue culture to determine the long-term effects of *FLCN* knockdown. To compare short-term effects, cells were grown for <2 months after *FLCN* knockdown. To investigate whether long-term *FLCN* deficiency increases tumorigenicity, we compared the growth of these *FLCN*-knockdown HK2 cells in soft agar (Fig. 1A). Short-term *FLCN* knockdown did not alter colony formation, while long-term *FLCN* knockdown led to markedly larger colonies, with a mean diameter >100 μm. Colony diameters within the long-term *FLCN* knockdown population were more divergent, indicating heterogeneity in colony formation.

To further assess anchorage-independent growth, these HK2 cells were grown as self-aggregated spheroids (Fig. 1B). We observed greater tumorigenicity after long-term *FLCN* knockdown than after short-term *FLCN* knockdown. We next analyzed the spheroid growth of additional nontarget and *FLCN* shRNA-knockdown clones (Supplementary Fig. 1), which supported our finding that long-term *FLCN* knockdown cells presented the greatest increase in spheroid diameter. These data revealed that long-term *FLCN* knockdown has a greater capacity to promote tumorigenicity than short-term *FLCN* knockdown.

### *FLCN* knockdown dramatically altered the transcriptome profile over time, as indicated by gene enrichment of cell cycle genes

**Fig. 2:**
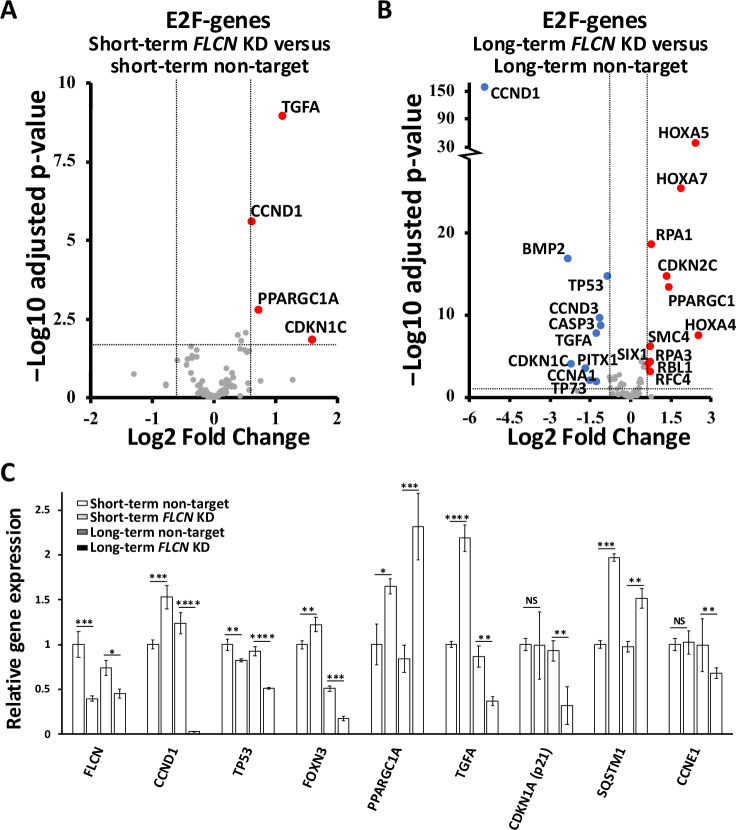
Differentially expressed genes linked to E2F and known FLCN regulatory genes after *FLCN* knockdown. Differential gene expression of E2F genes was compared between **A** short-term *FLCN* knockdown cells and nontarget shRNA knockdown cells and **B** long-term *FLCN* knockdown and nontarget shRNA knockdown HK2 cells after RNA sequencing. Volcano plots are shown with the following thresholds: ≤ & ≥ 2.5-fold change and padj < 0.05 with false rate discovery (FDR) correction applied. **C** FLCN-linked genes from this RNA sequencing experiment are graphed and include *FLCN*, *CCND1*, *TP53*, *PPARGC1A*, *TGFA*, *CDKN1A*, *SQSTM1* and *CCNE1*.

Changes in transcription between nontarget HK2 cells and HK2 cells with *FLCN* knockdown were investigated. A PCA and Euclidean cluster dendrogram plot of DESeq2-normalized samples showed that the experimental replicates clustered tightly into distinct groups. The most significant factor of interest (PC1) separated the long-term *FLCN* knockdown group from all the other groups (Supplementary Fig. 2A, B). While short-term *FLCN* knockdown altered the expression of 216 genes, this number of genes strikingly increased to 1063 after long-term *FLCN* knockdown (Fig. 1C; ≥ 2.5-fold change up or down, padj < 0.05 with false rate discovery (FDR) correction applied). We also observed that ageing alone altered 250 gene transcripts by at least two-fold in the control cells, but 3.4 times more genes had altered gene expression when *FLCN* loss was combined with ageing. A WNT signaling signature was evident after both short- and long-term *FLCN* knockdown. The DEGs associated with long-term *FLCN* knockdown showed the greatest enrichment in ‘Aberrant regulation of mitotic G_1_/S transition in cancer due to RB1 defects’, Reactome: R-HSA-9659787 ( > 100-fold enrichment and 7.65E-04 FDR correction applied). Therefore, we investigated the involvement of RB1/E2F-regulated genes in S-phase entry. When comparing short-term *FLCN* knockdown to its wild-type control (Fig. 2A), *TGFA*, *CCND1*, *PPARGC1A* (also known as *PGC1a*) and *CDKN1C* were upregulated with *FLCN* knockdown. After long-term *FLCN* knockdown, many additional genes were differentially expressed. The three *HOX* genes exhibited the greatest increase in expression, while *TP53*, *BMP2*, *TGFA* and *CCND1* were downregulated (Fig. 2B). Changes in the expression of the E2F-regulated genes *CCND1, TP53*, *PPARGC1A*, *TGFA*, *c-Jun*, *RPA1* and *RBL1* [25] are shown in Fig. 2C, as are *p21* (*CDKN1A*) and *FOXN3*, which can arrest cells at the G_1_ checkpoint [26, 27]. The mRNA expression levels of *CCND1*, *TP53*, *FOXN3*, *p21* (*CDKN1A*), *TGFA* and *CCNE1* were downregulated after long-term *FLCN* knockdown. In support of these observed alterations in cell cycle regulatory components, compared with wild-type cells, *FLCN* knockdown cells presented small but significant changes in 2 N (G_0_/G_1_) and 4 N (G_2_/M) expression according to flow cytometry (Supplementary Fig. 2C).

**Fig. 3:**
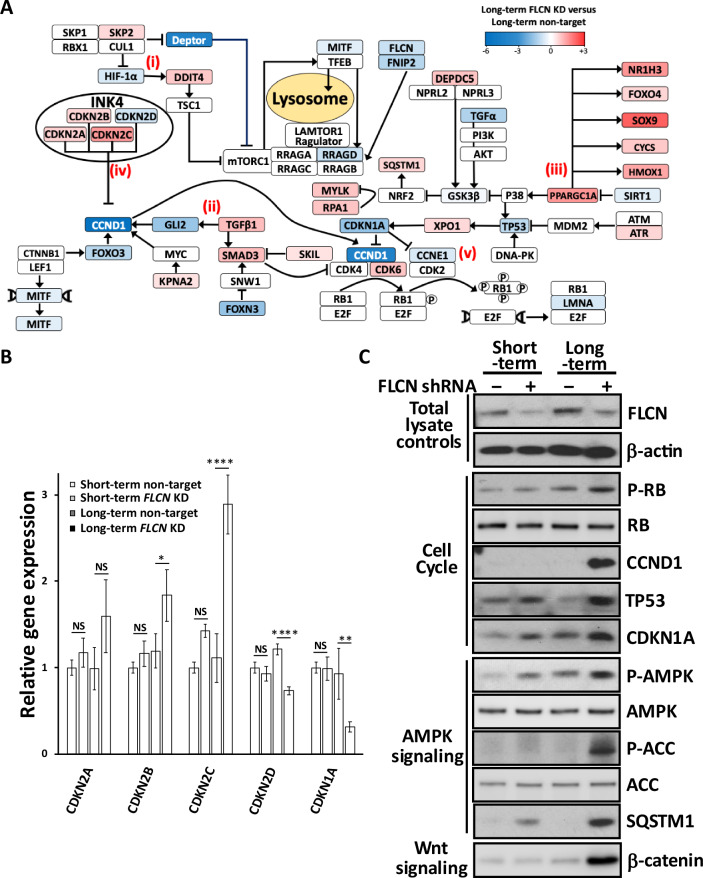
Differentially expressed genes and their associated signaling pathways. **A** RNA sequencing was used to compare long-term *FLCN* shRNA-mediated knockdown and nontargeting shRNA-mediated knockdown in HK2 cells (scale is set to log2-fold change). Each gene is depicted in a signaling flow diagram. The known signaling functions of FLCN include (i) enhancing HIF-1α activity, as shown by increased expression of the HIF-1α target genes *SLC2A2* and *REDD1*. A signaling feedback mechanism reduces HIF-1α through reducing HIF-1α expression and upregulating *SKP2*, a HIF-1α inhibitor. (ii) Upregulation of TGFβ/SMAD3 signaling upon knockdown of *FLCN*. SMAD3 is an inhibitor of the CCND1-CDK4/CDK6 complex. (iii) The expression of PGC1α and its downstream genes *NR1H3*, *FOXO4*, *SOX9*, *CYCS*, and *HMOX1* was markedly upregulated upon *FLCN* knockdown. (iv) The INK4 family members that inhibit *CCND1* and *CDKN2A-C* are increased, while *CDKN2D* is reduced. (v) Reduced expression of *CDKN1A* inhibits both CCND1 and CCNE1 activity. This finding demonstrated the presence of a transcriptional feedback mechanism that enhances CDK4/6 and CDK2 activity. Further differences were observed with enhanced *CDK6* expression and decreased expression of *CCND1* and *CCNE1*. **B** Relative gene expression of cyclin-dependent kinase inhibitors across the different HK2 cell lines was calculated (*n* = 3). **C** Protein lysates from spheroids generated from HK2 cells with or without short- or long-term *FLCN* shRNA knockdown (nontargeted shRNA was used as a control) were probed for phosphorylated RB1, CCND1, TP53, and CDKN1A; phosphorylated AMPK; and ACC; SQSTM1, β-catenin, FLCN, and β-actin, which were used as loading controls (*n* = 3).

To determine dysregulation at the pathway level, we assessed changes in cell signaling after *FLCN* knockdown (Fig. 3A). After long-term *FLCN* knockdown, we observed marked alterations in the expression of genes involved in multiple pathways that have been previously linked to BHD: (i) upregulation of *HIF1A* [12] toward the gene targets SLC2A2 (11.2-fold) and DDIT4 (1.6-fold) and the dramatic reduction of DEPTOR (by 93%), the negative regulator of mTORC1. (ii) Increased expression of TGFβ (2.1-fold increase) and SMAD3 (1.8-fold increase) was detected, suggesting elevated TGFβ-SMAD signaling [28]. (iii) There was a 20% reduction in SIRT1 expression (a negative regulator of PPARGC1A), a marked 2.7-fold increase in *PPARGC1A* expression, and a subsequent increase in PPARGC1A-regulated genes, namely, *NR1H3*, *SOX9*, and *HMOX1* ( > 2.5-fold change and <0.0001 adjusted p value). This observation supports previously published work in which *PPARGC1A* was observed to drive mitochondrial biogenesis and metabolic transformation in BHD cell models [11]. More than half of the G_1_/S regulatory genes were dysregulated following *FLCN* knockdown, with notable changes to (iv) INK4 family members that negatively regulate CCND1 (graphed in Fig. 3B, CDKN2A-D) and (v) cell cycle regulators that control RB1 phosphorylation and E2F activation. We also found evidence that *FLCN* knockdown cells employ feedback mechanisms to reduce the expression of G_1_/S regulatory components. For example, although CCND1 was initially upregulated upon *FLCN* knockdown (Fig. 2C and Supplementary Fig. 4), *CCND1* mRNA was strikingly downregulated by 97% after long-term *FLCN* knockdown (Figs. 2C and 3A). Given the bidirectional relationship between metabolism and cell cycle progression [29], we speculate that the altered metabolism observed in *FLCN*-deficient cells [11, 12] could underlie the defects in E2F-regulated cell cycle gene expression that become more pronounced and protumorigenic over time.

To assess whether alterations in spheroid growth correlated with the observed alterations in cell cycle pathway dysregulation, we generated lysates from the spheroids shown in Fig. 1B and analyzed a panel of cell cycle proteins. We found that spheroids from long-term *FLCN* knockdown had elevated CCND1 protein expression and RB1 phosphorylation (Fig. 3C). These alterations after long-term *FLCN* knockdown could correlate with their increased growth properties. As negative regulators of cyclin:cyclin dependent kinase (CDK) complexes, we analyzed TP53 and cyclin-dependent kinase inhibitor 1 A (CDKN1A, known as p21/WAF1). We found that these genes were highly expressed following *FLCN* knockdown, and in both cases, the levels were greater after long-term knockdown. These findings contrast with the RNA data, in which *CCND1*, *TP53* and *CDKN1A* mRNA expression was downregulated in response to long-term *FLCN* knockdown (Fig. 2C). Long-term *FLCN* knockdown also elevated the expression of β-catenin, which is associated with increased WNT signaling, in line with our RNA sequencing findings (Fig. 1C). Sequestosome 1 (SQSTM1, also known as p62) mRNA was also increased by 1.5-fold (Fig. 2C), and protein expression was markedly enhanced (Fig. 3C). AMPK and acetyl-CoA carboxylase (ACC) phosphorylation was enhanced following *FLCN* knockdown and was more evident after long-term knockdown (Fig. 3C). High levels of SQSTM1 and AMPK activation are consistent with metabolic alterations and energy stress, as previously reported in BHD models [11].

### FLCN interacts with components of the cell cycle and DDR

**Fig. 4:**
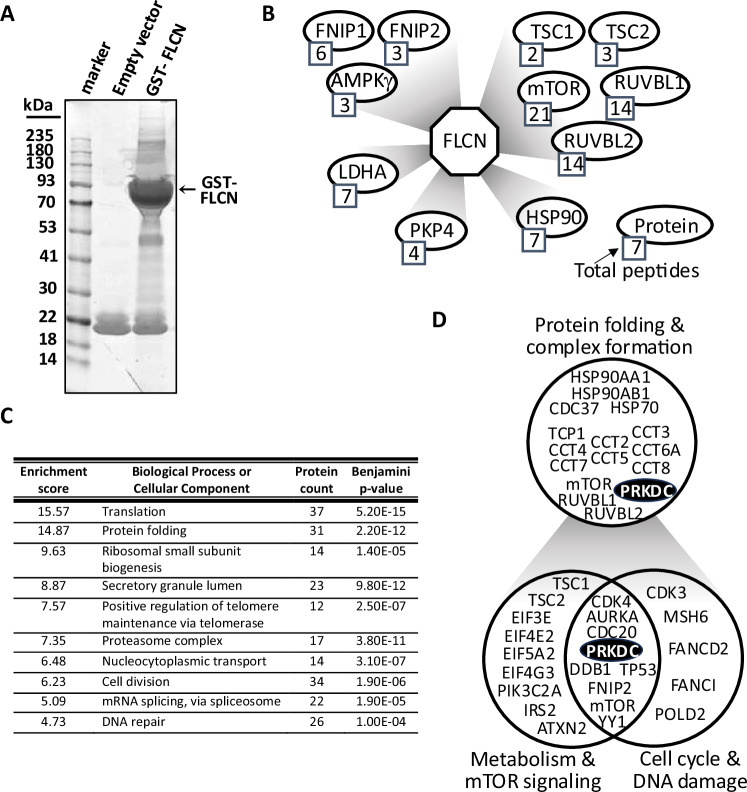
FLCN has an extensive PPI network. **A** GST-FLCN was overexpressed in HEK293 cells, after which the purified and interacting proteins were separated via SDS‒PAGE and stained with colloidal blue. The gel was sectioned for mass spectrometry analysis. **B** The FLCN protein interaction network is shown, which includes known FLCN interactions. The total peptide sequences identified after mass spectrometry are indicated. **C** FLCN-binding proteins identified by mass spectrometry were analyzed by DAVID, and the top ten enriched scored biological processes or cellular components are presented. **D** FLCN-binding proteins are involved in protein folding and complex formation, metabolism and mTOR signaling, and cell cycle and DNA damage.

To further define FLCN as a tumor suppressor, mass spectrometry of GST-tagged FLCN-interacting partners was performed (Fig. 4A). This revealed 603 potential FLCN-binding proteins, and successful enrichment of FLCN-binding proteins was confirmed via the identification of previously characterized FLCN interactors, such as FLCN-interacting protein 1 (FNIP1) [30], FNIP2 [31], LDHA [13], PKP4 [14, 15] (Fig. 4B) and the BRCA1 A complex component BRE [32]. When considering identified interactors for which more than one unique peptide was identified, 541 potential FLCN-binding proteins were mapped to functional clusters by DAVID analysis (Fig. 4C). This revealed that the strongest enrichment was in proteins involved in translation, in keeping with the known function of FLCN in the mTORC1 pathway. Interestingly, we identified protein folding chaperones as highly enriched in our dataset; these chaperones included all eight components of the TRiC/CCT chaperonin complex and two isoforms of HSP90 plus the co-chaperone CDC37. The chaperonin CCT helps fold both mLST8 and Raptor, which are part of mTORC1 [33], while the R2TP complex (which includes RUVBL1 and RUVBL2), together with heat shock protein 90 (HSP90), is a chaperone for the assembly of protein complexes, including phosphatidylinositol 3-kinase (PI3K)-like kinases (PIKKs), such as TOR and PRKDC (refered to as DNA-dependent protein kinase (DNA-PK)) (highlighted in Figs. 4B and 4D) [34]. Additionally, FLCN was previously described as an HSP90 client protein, where the HSP90-FLCN interaction enhances the stability of FLCN, while the FLCN binding partners FNIP1/FNIP2 function as co-chaperones [35]. Several CCT substrates are cell cycle proteins, particularly those involved in the G_1_/S phase (discussed in [36]), so it was interesting to observe that cell division was also one of the top 10 enriched processes (Fig. 4C). There was a greater proportion of FLCN-binding proteins linked to the G_1_/S phase in the different stages of the cell cycle (Supplementary Fig. 3A), including CDK1, CDC20, TP53 and RB-binding protein 7 (RBBP-7). These findings support our RNA sequencing findings, indicating that FLCN is linked to the G_1_/S phase of the cell cycle. One cell cycle protein, DNA-PK, was our top interactor, with the most peptides identified (Supplementary Fig. 3B). This protein also plays a role in DNA repair, another process enriched in our interactome (Fig. 4C). Indeed, a number of interactors were involved in metabolism, the cell cycle and DNA damage (Fig. 4D).

### DNA-PK interacts with FLCN

**Fig. 5:**
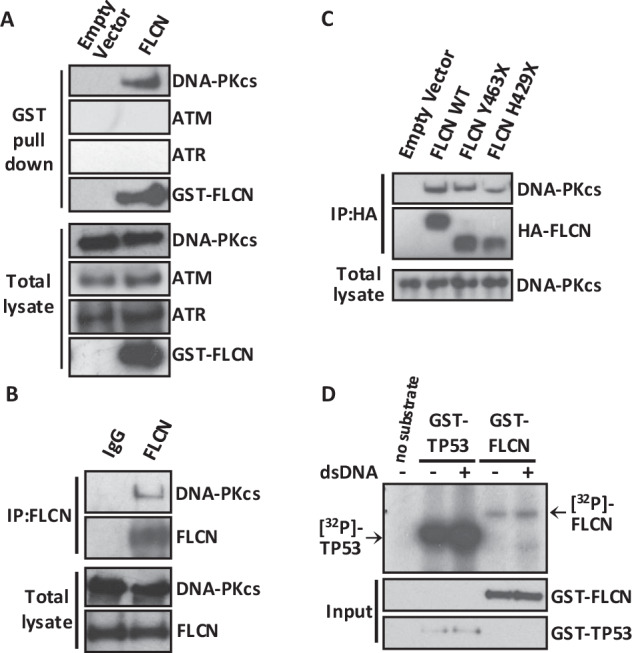
FLCN interacts with DNA-PK. **A** GST-tagged FLCN was overexpressed in HEK293 cells and used as a bait protein to validate the interactions between FLCN and endogenously expressed DNA damage components (DNA-PKcs, ATM, and ATR) via GST pull-down. **B** Endogenous FLCN was immunoprecipitated using an antibody against N-terminal FLCN, and the interaction of endogenous DNA-PKcs was detected via western blotting. **C** HA-tagged FLCN constructs (wild-type FLCN (WT)) and two patient-derived C-terminal truncation mutants (Y463X and H429X) were overexpressed in HEK293 cells and immunoprecipitated using anti-HA antibodies, and bound endogenous DNA-PKcs was detected via western blotting. **D** DNA-PK kinase assays were performed using GST-FLCN or GST-TP53, which were overexpressed and purified from HEK293 cells. The incorporation of radiolabeled phosphate [^32^P] was determined with active DNA-PK, which was further induced by supplementation with short double-stranded DNA (dsDNA).

As our top interactor, with 66 unique peptides and 17.9% coverage (Supplementary Fig. 3B, C), we focused our attention on DNA-PKcs. DNA-PKcs plays an important role in cell cycle check point control during DNA damage [37], so this aligns with our transcriptomic work linking FLCN to cell cycle dysregulation (Fig. 2B) and the finding of DNA-PK in our FLCN interaction network (Fig. 4D). Given that there are limitations to overexpressed protein purification methods, i.e., potential nonspecific or artificial protein binding to either beads or mislocalized overexpressed proteins, we carried out further FLCN-binding validation experiments on DNA-PKcs. We initially examined whether endogenous DNA-PKcs copurified with GST-tagged FLCN (Fig. 5A). We observed a robust interaction between DNA-PKcs and GST-FLCN but not between DNA-PKcs and the empty vector control, revealing that there was no nonspecific binding of DNA-PKcs to the beads. The other PIKK family members, ATM and ATR, were not immunoprecipitated with FLCN. After further validating the DNA-PKcs interaction, we observed the interaction of endogenous DNA-PKcs with immunoprecipitated endogenous FLCN (Fig. 5B). Next, we compared the associations of DNA-PKcs with wild-type FLCN and the BHD patient-derived mutants Y463X and H429X (Fig. 5C). Both C-terminal truncation mutants of FLCN interacted with endogenous DNA-PKcs. We then considered that FLCN might be a direct substrate of DNA-PKcs. Therefore, in vitro DNA-PK kinase assays were performed using TP53 as a DNA-PK substrate control (Fig. 5D). Supplementation with dsDNA was used to further enhance the kinase activity of DNA-PK. Unlike that of TP53, where DNA-PK-mediated phosphorylation was enhanced with the supplementation of dsDNA, we observed a much weaker level of [^32^P]-incorporation into FLCN. Neither FNIP1 nor FNIP2 further enhanced this low level of phosphorylation (data not shown). Given the low levels of [^32^P]-incorporation into FLCN, which was not further enhanced by supplementation with dsDNA, FLCN is unlikely to be a direct substrate of DNA-PK in these assays.

### Cell cycle progression is dysregulated following long-term loss of FLCN

**Fig. 6:**
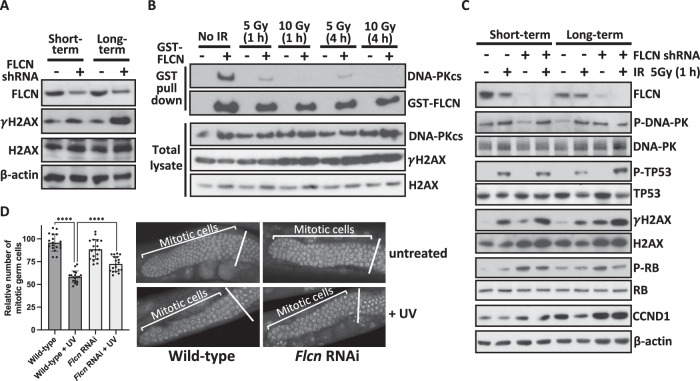
*FLCN* knockdown promotes cell cycle progression. **A** Serine 139 phosphorylation of the histone variant H2AX (γH2AX) was assessed under basal conditions in HK2 cells grown in standard cell culture with and without short-term or long-term *FLCN* shRNA knockdown (nontargeted shRNA was used as a control). **B** The FLCN/DNA-PKcs interaction was investigated following IR-induced DNA damage. GST-tagged FLCN was overexpressed in HEK293 cells that were then subjected to IR (5 or 10 Gy) and left for either 1 or 4 h prior to cellular lysis, as indicated. GST-FLCN was purified, and endogenous DNA-PKcs was detected via western blotting. **C** In HK2 cells with or without short- or long-term *FLCN* shRNA knockdown, DNA damage markers were assessed following 5 Gy IR for 1 h, where indicated. γH2AX, P-DNA-PKcs (Ser2056) and the downstream DNA-PK substrate P-TP53 (Ser15) are shown. G_1_/S phase cell cycle markers CCND1 and P-RB1 were analyzed and FLCN and β-actin were used as controls. **D** Mitotic germ cells were quantified in *C. elegans* with and without *Flcn* siRNA knockdown and subjected to UV damage, where indicated. Mitotic germ cells were scored and graphed. Representative images of the mitotic cells are presented (*n* = 18).

As DNA-PKcs regulates the phosphorylation of H2AX in response to cell cycle progression and DNA damage [38], we next analyzed γH2AX (H2AX phosphorylated at Ser139). Following short-term *FLCN* knockdown, γH2AX expression was enhanced, and long-term *FLCN* knockdown significantly elevated γH2AX expression (Fig. 6A and Supplementary Fig. 4A). γH2AX is classically regarded as a marker of DNA damage involved in the surveillance and repair of double strand breaks, such as those induced by ionizing radiation (IR). However, γH2AX has also been reported to occur independently of double-strand DNA breaks [39], in mitotic cells [38] and in response to serum starvation [40]. Therefore, we next assessed the interaction between DNA-PKcs and GST-FLCN following IR and found that endogenous DNA-PKcs dissociated from GST-FLCN after IR treatment at 5 and 10 Gy (Fig. 6B). This finding revealed that the FLCN/DNA-PK interaction is regulated by DNA damage. DNA damage markers in 5 Gy IR-treated cells were analyzed to determine whether *FLCN* knockdown altered DDR signaling. IR enhanced γH2AX expression, as expected, as indicated by increased basal and IR-induced γH2AX expression in *FLCN*-deficient HK2 cells (Fig. 6C). DNA-PKcs autophosphorylation is essential for the appropriate regulation of DNA strand end processing, enzyme inactivation, and complex dissociation from DNA (see review [41]). However, no change in DNA-PK autophosphorylation was observed upon *FLCN* knockdown. While phosphorylation of TP53 at Ser15 was induced under IR, there was no difference between *FLCN* knockdown and wild-type cells (Fig. 6C). These data showed that although γH2AX is elevated following *FLCN* loss, *FLCN* loss has no direct impact on IR-induced DNA-PK signaling; i.e., elevated γH2AX is unlikely to be reflective of double-strand DNA breaks. When the RNA sequencing data with and without *FLCN* knockdown were analyzed through Mutect2, there was no evidence of enhanced DNA mutation (data not shown). This finding indicates that *FLCN* loss is unlikely to enhance DNA damage but might be related to cell cycle control linked to DNA-PK.

To determine whether cell cycle defects following *FLCN* loss are linked to the G_1_/S phase transition in mammalian cells, we examined the expression of CCND1 in HK2 cells following IR treatment (Fig. 6C). CCND1 expression was greater after *FLCN* was knocked down, which is in line with the findings of previous work [17]. After long-term *FLCN* knockdown, CCND1 protein expression further increased (Fig. 6C), even though *CCND1* mRNA was substantially reduced (Supplementary Fig. 4B). In healthy cells, CCND1 protein levels are typically reduced after IR as part of a normal DNA damage cell cycle checkpoint control mechanism [41]. However, in *FLCN* knockdown cells following IR treatment, CCND1 protein levels remain elevated, indicating a defect in the normal control of CCND1 after DNA damage (Fig. 6C). Additional HK2 clones with *FLCN* knockdown showed higher levels of CCND1 when compared to control HK2 cells (Supplementary Fig. 4C). Increased RB1 phosphorylation was also observed after *FLCN* knockdown, implying an increase in the number of active G_1_/S cyclin-CDK complexes (Fig. 6C). RB1 phosphorylation leads to E2F activation and entry into S phase, suggesting that knockdown of *FLCN* favors G_1_/S checkpoint slippage. In support of this observation, transcriptomic analysis revealed enrichment of E2F-regulated genes in *FLCN*-knockdown HK2 cells (Fig. 2B).

To examine the role of FLCN in an alternative system, we analyzed *C. elegans* with and without *Flcn* RNAi knockdown. *C. elegans* has previously been used as a model organism to characterize the effects of DNA damage and cell cycle checkpoint control in germ cells (reviewed in [42]). While we observed no change in the relative number of mitotic germ cells upon *Flcn* knockdown, *Flcn*-knockdown worms were defective in cell cycle arrest caused by UV-induced DNA damage. We observed a small increase in the relative number of mitotic germ cells after UV irradiation (Fig. 6D). These findings support the hypothesis that *Flcn* knockdown results in dysregulation of cell cycle progression following DNA damage. Overall, *FLCN* knockdown leads to cell cycle dysregulation with and without DNA damage, where we observed heightened levels of CCND1 and RB1 phosphorylation as well as enhanced E2F-mediated transcription of S-phase genes.

## Discussion

Despite the germline *FLCN* mutation, RCC takes several decades to develop in BHD patients, but most studies to date have analyzed only the immediate impact of *FLCN* loss in cells. Therefore, we explored the long-term consequences of *FLCN* loss through transcriptomic and proteomic analysis to better characterize FLCN as a tumor suppressor and, in doing so, uncovered links to DNA-PK and cell cycle control.

Proteomic analysis revealed that FLCN interactors are involved in protein folding, the cell cycle and DNA repair, including DNA-PKs. DNA-PKcs showed strong binding to both wild-type and BHD patient-derived FLCN mutants. Arguing against a nonspecific binding artifact, this interaction was completely reversed under IR. DNA-PK plays a key role in the DNA damage response. *FLCN* loss was previously linked to DNA damage, where *FLCN*-deficient cells were shown to be sensitive to PARP inhibitors due to impairment of the DNA repair ability associated with the BRCA1 complex [32]. In our study, we detected the BRCA1 A complex member BRE in our FLCN pulldown experiments and observed that long-term *FLCN* knockdown resulted in an increase in γH2AX. We believe that this increase in γH2AX expression is unlikely to be due to DNA damage, as other markers of DNA repair signaling were unaltered. DNA-PKs play roles beyond the DNA damage response, including in cell cycle progression [43]; therefore, we propose that *FLCN* loss causes defects in cell cycle control. It is likely that the higher levels of γH2AX after *FLCN* knockdown reflect the known role of DNA-PK in enhancing γH2AX expression during cell cycle control, which can occur independently of DNA damage [43]. Therefore, the large transcriptional changes in cell cycle genes observed upon *FLCN* knockdown are likely due to epigenetic alterations or altered gene expression downstream of metabolic signaling changes rather than an accumulation of unrepaired DNA errors.

While our study specifically focused on the G_1_/S phase of the cell cycle, it should be noted that FLCN has also been linked to the G_2_/M phase [44]. Upon *FLCN* knockdown, we observed aberrant protein expression of CCND1. Previous work revealed that the translation of *CCND1* mRNA was negatively regulated by a FLCN regulatory cis-acting element in its 3’-untranslated region [17], which partially helps to explain why CCND1 protein levels increase upon *FLCN*-deficiency. We observed that CCND1 protein levels remained elevated even after IR-induced DNA damage in the absence of FLCN, suggesting that the control of DNA damage at the cell cycle checkpoint may be compromised by FLCN knockdown. As RB1 phosphorylation was elevated upon *FLCN* knockdown, there must be a higher level of CDK activity toward RB1 in these cells. Analysis of the RNA sequencing data provided further evidence of RB1/E2F activation, as E2F target genes were more highly expressed in the absence of *FLCN*. Furthermore, upon *FLCN* knockdown, we observed a modest reduction in the G_1_ population of cells, suggesting a potential increase in proliferative drive through G_1_/S in the absence of *FLCN*. The high protein levels of CCND1 and RB1 hyperphosphorylation in long-term *FLCN*-knockdown HK2 tumor spheroids likely contributed to their observed tumorigenicity.

Our interactome data and enrichment of protein folding chaperones are consistent with previous observations that FLCN is folded and assembled into large multiprotein complexes. Recent publications, such as the FLCN-FNIP2-Rag-Ragulator complex [45] and the mTORC1-TFEB-Rag-Ragulator megacomplex, which includes the FLCN-regulated RagC [46], have shown that FLCN is involved in megacomplexes that control metabolism.

While a limitation of this work is that our cell line data is restricted to the HK2 cell line due to the resources required for long-term culturing, we also used a *C. elegans* model to further confirm that FLCN is connected to cell cycle control. The DDR pathway is defined by several key features, one of which is cell cycle checkpoint control, which is regulated by DNA-PK. This helps prevent the propagation of somatic mutations into progeny cells when DNA damage is present and not repaired. Two pieces of evidence indicate a defect in proper cell cycle checkpoint control upon *FLCN* loss. First, *C. elegans* animals with *Flcn* knockdown had more mitotic germ cells following DNA damage than did those with nontarget *Flcn* knockdown. Second, in mammalian HK2 cells, CCND1/RB1-P remained elevated in the presence of DNA damage when *FLCN* was knocked down, while this checkpoint was engaged normally in wild-type cells (as observed by reduced CCND1/RB1 phosphorylation). Further studies in additional cell models and systems would help confirm and expand these findings to explain whether such dysregulation of cell cycle occurs over time and drives earlier onset of cancer progression observed in BHD patients.

## Materials and methods

### Cell culture

Unless stated otherwise, laboratory reagents were purchased from Merck Millipore (Burlington, Massachusetts, USA). HEK293 cells were purchased from American Type Culture Collection (Manassas, Virginia, USA). Cell maintenance and the generation of stable *FLCN*-knockdown HK2 cells have been described previously [10] and all clones were confirmed to match the HK2 line by STR analysis and knockdown clones remained stable in culture (outsourced to Northgene, Deeside, UK). Short- and long-term knockdown were defined as < 30 and > 105 passages after clonal selection, respectively. Lipofectamine 2000 transfection was carried out according to the manufacturer’s protocol (Life Technologies, Paisley, UK). Irradiation (IR) was carried out using a Gammacell 1000 Elite (Nordion Gamma Technologies, Abingdon, UK). The cells were checked with a Venor™ GeM Advance Mycoplasma Detection Kit (Minerva Biolabs, Berlin, Germany) according to the manufacturer’s guidelines and were found to be mycoplasma negative. Tumor spheroid growth assays in soft agar were performed as previously described [47]. For spheroid protein analysis, 12,500 cells were allowed to self-aggregate in 1.5% (w/v) agarose-coated wells. Forty-eight spheroids per cell line were lysed in 1x sample buffer (62.8 mM Tris, 10% (v/v) glycerol, 2% (w/v) SDS, 0.1% (w/v) bromophenol blue, 50 mM dithiothreitol (DTT) added just before use), sonicated and boiled prior to blotting. Cell lysis and western blot analysis were performed as previously described [48].

### Antibodies

Unless otherwise specified, the antibodies used were purchased from Cell Signaling Technology (Danvers, MA, USA). The phospho-specific antibodies used included p-H2AX Ser139 (#9718 P (20E3)), p-TP53 Ser15 (#9286 P (16G8)), p-RB1 Ser780 (D59B7), p-AMPK Thr172 (#2531), p-ACC Ser79 (#3661) and p-DNA-PK Ser2056 (ab124918 from Abcam Plc., Cambridge, UK). The pan-antibodies used included β-actin (#84573 (D6A8)), CCND1 (#2922), CDKN1A (12D1), SQSTM1 (#5114), and TP53 (DO-1 from Bethyl Laboratories Ltd., Montgomery, TX, USA) and from Merck Millipore both GST (#DAM1411332), ATM (#2873 (D2E2)), ATR (#2790), DNA-PKcs (#D00148436), and HA (#1186742300 from Roche Products Limited, Welwyn Garden City, UK). The custom-made N-terminal anti-FLCN was previously described [10].

### GST pull-down and coimmunoprecipitation assays

HEK293 cells were transfected with either GST-FLCN in the pDEST27 backbone (Life Technologies, 11812013) or the pcDNA3 empty vector. Then, 150 µL of lysate was incubated with preblocked glutathione-Sepharose 4B beads (GE Healthcare, Chalfont St. Giles, UK) at 4°C in a rotary shaker for 3 h. The beads were washed 5 times in BHD lysis buffer [8] supplemented with 300 mM NaCl for DNA-PKcs (250 mM for all other proteins). The bound proteins were eluted using 10 mM glutathione in elution buffer (20 mM HEPES (pH 8), 200 mM NaCl, 5 mM MgCl_2_) to avoid elution and subsequent detection of proteins that bound nonspecifically to the beads. The eluent was mixed with NuPAGE LDS sample buffer containing 100 mM DTT. For coimmunoprecipitation, HEK293 cells were transfected with HA-FLCN wild type, HA-FLCN Y463X or HA-FLCN H429X in the pcDNA3.1 vector or pcDNA3.1 empty vector (described in [8]). The lysates were precleared with unblocked protein G-Sepharose beads (GE Healthcare) for 1 h at 4°C and then centrifuged for 3 min at 3000 rpm and 4°C to remove the beads. The lysates were then incubated with anti-HA antibodies for 2 h at 4°C in a rotary shaker before BSA-blocked protein G-Sepharose beads were added for 2 h. The beads were subsequently washed, and the proteins were dissociated with sample buffer containing 25 mM DTT at 70°C for 10 min.

### Mass spectrometry sample preparation, sequencing, and FLCN protein–protein interaction analysis

Proteins were identified from gel fragments by liquid chromatography-tandem mass spectrometry (LC‒MS/MS), as previously described [49]. The identified proteins were filtered by removal of keratins. Proteins with 2 or more unique peptides identified were analyzed using the Database for Annotation, Visualization and Integrated Discovery (DAVID) Functional Annotation Clustering [50]. Known FLCN interactors were screened manually using the following GO terms: (i) protein folding GO:0006457, (ii) metabolic processes GO:0031323, (iii) cell cycle GO:0007049 and (iv) response to DNA damage GO:0006974.

### Statistical analysis

Unless stated otherwise, after determining a normal (Gaussian) distribution (Shapiro–Wilk test), ordinary one-way ANOVA with Tukey’s multiple comparisons was carried out using Prism GraphPad; * *p* < 0.05, ** *p* < 0.01, *** *p* < 0.001, **** *p* < 0.0001, and not significant (ns). All the data are presented as the mean ± SEM. Experiments were repeated 3 times as biological repeats, unless stated otherwise. Figure legends state *n*-numbers and statistical analysis if different to the above. No inclusion or exclusion criteria, randomization or blinding of experiments were carried out for either the cell line and *C. elegan* experiments.

## Supplementary information

Supplementary Material

Supplementary Figure 1

Supplementary Figure 2

Supplementary Figure 3

Supplementary Figure 4

Uncropped western blots

## Data Availability

Proteomic dataset is available at 10.6084/m9.figshare.25943011. RNA sequencing data is available at Gene Expression Omnibus, accession GSE283021.
