# Supplementary material for: Characterizing the tumor suppressor activity of FLCN in Birt-Hogg-Dubé syndrome cell models through transcriptomic and proteomic analysis
Source: Oncogene. 2025 Mar 25;44(23):1833–43. doi: 10.1038/s41388-025-03325-z (PMC12143978; doi:10.1038/s41388-025-03325-z)
Supplement: Supplementary file 1 — Supplementary Material [file 41388_2025_3325_MOESM1_ESM.docx]

**Supplementary Material and Methods**

*RNA sample preparation, sequencing, and analysis*

RNA was prepared for sequencing as described previously [1], except the Illumina® TruSeq® RNA sample preparation v2 (Illumina Inc.) kit was used for library preparation. Libraries were normalized to 8 nM and pooled together and sequenced on the Illumina® MiSeq with a 150 cycle, version 3, cartridge according to the manufacturer’s instructions. Reads were quality trimmed with FastP [2] before mapping with STAR (2-Pass) [3] and counts assigned with FeatureCounts [4]. Differentially expressed transcripts were identified using DeSeq2 package in R [5]in the SARTools wrapper. Analysis was carried out on all pairwise comparisons in the dataset. P-values were corrected for multiple testing using the Benjamini–Hochberg false discovery rate (FDR) method. DDR and cell cycle gene lists were obtained from AmiGo 2, GO: 0006974 (DDR), and GO: 0007049 (cell cycle) (http://amigo.geneontology.org/amigo). The E2F target gene list was taken from Bracken *et al*. [6]. Enrichment analysis was performed using REACTOME [7].

*In vitro DNA-PK kinase assay*

DNA-PK Kinase Enzyme System (#V4106, Promega, Chilworth, UK) was used. Following the manufacturer's protocol, 25 U/reaction of purified DNA-PK holoenzyme, 150 ng/reaction of either purified GST-FLCN or GST-TP53. The assay was activated with 100 mM ATP containing 1 μCi γ-[^32^P]. Reactions were incubated for 45 min at room temperature in the presence or absence of dsDNA. Kinase assay was stopped with the addition of 1x NuPage LDS sample buffer. Gels were vacuum-dried and γ-[^32^P] incorporation was measured by autoradiography.

*Flow cytometry*

DRAQ5™ (BioStatus) was added to live cell samples 10 min prior to analysis to quantify DNA content. The percentage of cells in G_1_ and G_2_ was quantified using Flow Jo v10 Software (Tristar).

*C. elegans strains and culture conditions*

*C. elegans* strains were cultured, maintained and synchronized according to the standard procedures [8] at 20 °C on nematode growth medium (NGM) agar plates seeded with OP50. Bristol N2 was used as the wild type strain. FNIP and FLCN RNAi clones were obtained from the Ahringer library available in Dr. Richard Roy’s laboratory (McGill University, Canada). The RNAi experiments were performed as described [9] and the control was bacteria transformed with empty vector. To perform the cell cycle arrest assays, young adult stage worms (42 h post-L1) treated with RNAi were exposed to 100 J/m2 UV-C irradiation using Stratagene UV crosslinker. 5 h post radiation, the number of mitotic germ cells in young adult hermaphrodites fixed in 100 % ethanol and stained with 4′,6-diamidino-2-phenylindole (DAPI) were counted using pictures obtained by fluorescence microscope (Zeiss Axioscope). For each condition in each experiment 15-20 animals were assessed.

**Supplementary References**

1. Johnson, CE, Dunlop, EA, Seifan, S, McCann, HD, Hay, T, Parfitt, GJ, *et al*. Loss of tuberous sclerosis complex 2 sensitizes tumors to nelfinavir-bortezomib therapy to intensify endoplasmic reticulum stress-induced cell death. Oncogene. 2018; 37:5913–5925.
2. Chen, S, Zhou, Y, Chen, Y, Gu, J. fastp: an ultra-fast all-in-one FASTQ preprocessor. Bioinformatics. 2018; 34:i884–i890.
3. Dobin, A, Davis, CA, Schlesinger, F, Drenkow, J, Zaleski, C, Jha, S, *et al*. STAR: ultrafast universal RNA-seq aligner. Bioinformatics. 2013; 29:15–21.
4. Liao, Y, Smyth, GK, Shi, W. featureCounts: an efficient general purpose program for assigning sequence reads to genomic features. Bioinformatics. 2014; 30:923–930.
5. Varet, H, Brillet-Guéguen, L, Coppée, JY, Dillies, MA. SARTools: A DESeq2- and EdgeR-Based R Pipeline for Comprehensive Differential Analysis of RNA-Seq Data. PLoS One. 2016; 11:e0157022.
6. Bracken, AP, Ciro, M, Cocito, A, Helin, K. E2F target genes: unraveling the biology. Trends Biochem Sci. 2004; 29:409–417.
7. Fabregat, A, Sidiropoulos, K, Viteri, G, Forner, O, Marin-Garcia, P, Arnau, V, *et al*. Reactome pathway analysis: a high-performance in-memory approach. BMC Bioinformatics. 2017; 18:142.
8. Stiernagle, T. Maintenance of C. elegans. WormBook. 2006; 1–11.
9. Kamath, RS, Martinez-Campos, M, Zipperlen, P, Fraser, AG, Ahringer, J. Effectiveness of specific RNA-mediated interference through ingested double-stranded RNA in Caenorhabditis elegans. Genome Biol. 2001;2:RESEARCH0002.

**Supplementary Figure Legends:**

**Supplementary Figure 1 Additional FLCN-knockdown clones exhibit increased tumorigenesis.** (**A**) Additional low- and high-passage *FLCN* knockdown (E1 and F5) and control (N4 and N8) clones were generated and plated under nonadherent conditions. Spheroids were imaged over 14 days (scalebar 200 μm), (**B**) with the % change in diameter compared to that on Day 0 graphed over time (n=37 across 3 biological repeats).

**Supplementary Figure 2: Differential gene expression clustering.** (**A**) Principal component analysis plot of DESeq2-normalized samples for long-term-WT (nontarget, purple), long-term-KD (*FLCN* knockdown, blue), short-term-WT (nontarget, yellow) and short-term-KD (*FLCN* knockdown, orange). The experimental replicates clustered tightly into distinct groups. The most significant factor of interest (PC1) separates long-term KD (*FLCN* knockdown) from other conditions. (**B**) A Euclidean cluster dendrogram plot of DESeq2-normalized samples showing sample similarity. (**C**) DNA content of HK2 wild-type (nontarget shRNA) and *FLCN* shRNA knockdown (short-term and long-term knockdown) cells was analyzed via flow cytometry using DRAQ7. The percentages of cells in the G_1_, S and G_2_ phases of the cell cycle are shown. Representative flow diagrams are presented (*n*=3).

**Supplementary Figure 3 FLCN interacts with components of the cell cycle and DNA damage apparatus.** (**A**) Cell cycle-related proteins identified in the FLCN interactome were plotted according to the cell cycle phase in which they function. (**B**) Potential FLCN-interacting proteins with known roles in the DNA damage response were identified, and the number of unique and total peptides was determined via mass spectrometry. (**C**) Unique peptides detected by mass spectrometry were mapped to PRKDC (DNA-PKcs) in yellow. These proteins represented 17.9% of the protein coverage. Underlined amino acids indicate a junction at which two unique peptides overlap.

**Supplementary Figure 4 Long-term FLCN knockdown causes a marked reduction in CCND1 mRNA expression.** (**A**) Relative levels of γH2AX (normalized to total H2AX) were assessed under basal conditions in HK2 cells grown in standard cell culture with and without short-term or long-term *FLCN* shRNA knockdown (nontargeted shRNA was used as a control) by densitometry (Image J), n=4. (**B**) QPCR for *CCND1* was carried out on HK2 cells with nontargeting shRNA and *FLCN* shRNA for short-term and long-term knockdown, using *ACTB* as a reference gene. The relative level of *CCND1* expression was normalized to 1 for the short-term nontargeting shRNA-knockdown HK2 cells (*n*=3). (**C**) Additional HK2 cells clones (N8 non-target; F5 and E1 FLCN knockdown), with or without short-term (S-T) or long-term (L-T) cell culture, were assessed following 5 Gy IR for 1 h for CCND1 protein expression. FLCN and β-actin serve as control.
