# Supplementary figures and images for: Characterizing the tumor suppressor activity of FLCN in Birt-Hogg-Dubé syndrome cell models through transcriptomic and proteomic analysis
Source: Oncogene. 2025 Mar 25;44(23):1833–43. doi: 10.1038/s41388-025-03325-z (PMC12143978; doi:10.1038/s41388-025-03325-z)

# Supplementary Figure 1

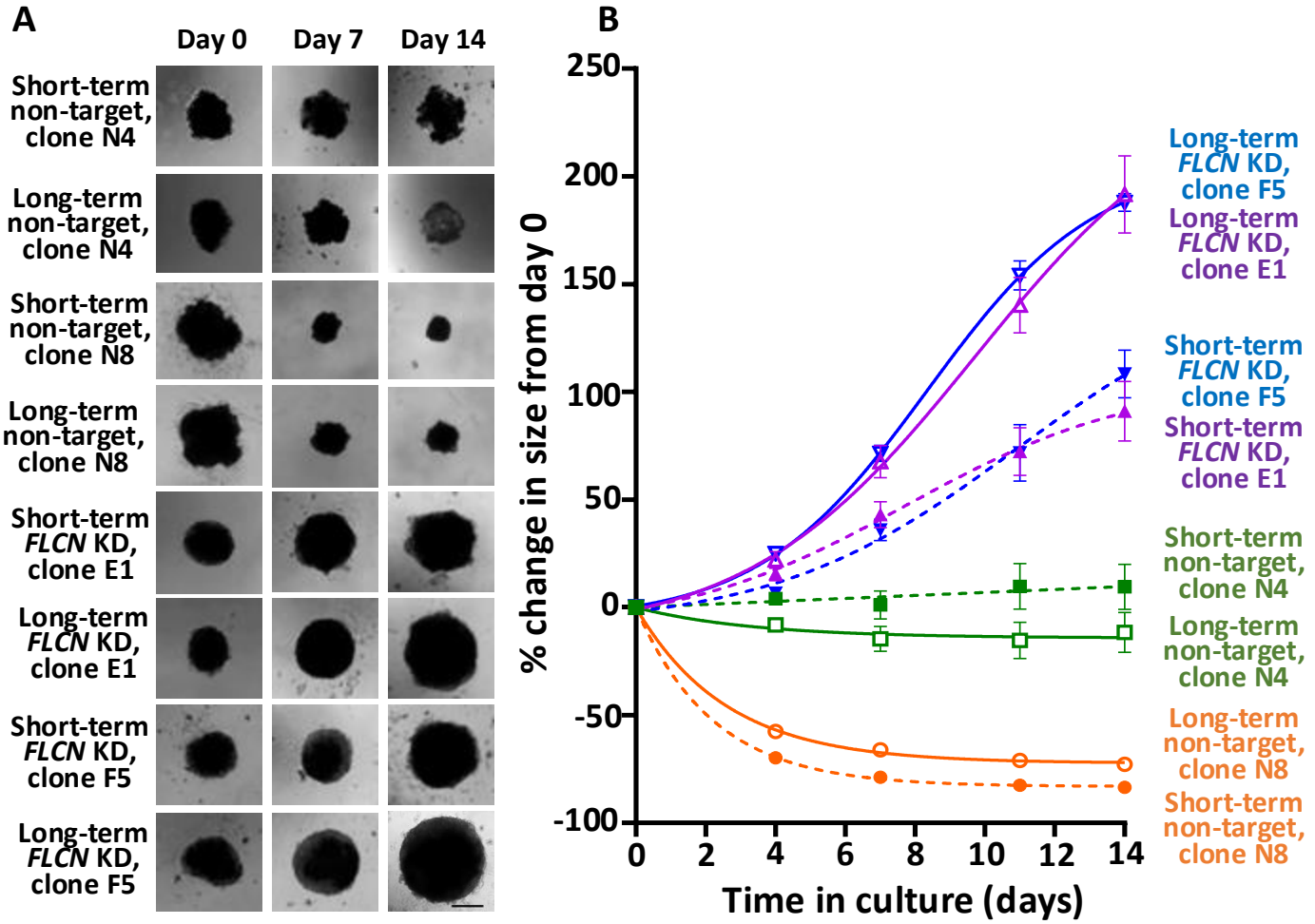

Supplement: Supplementary file 2 — Supplementary Figure 1 [file 41388_2025_3325_MOESM2_ESM.pdf]

# Supplementary Figure 2

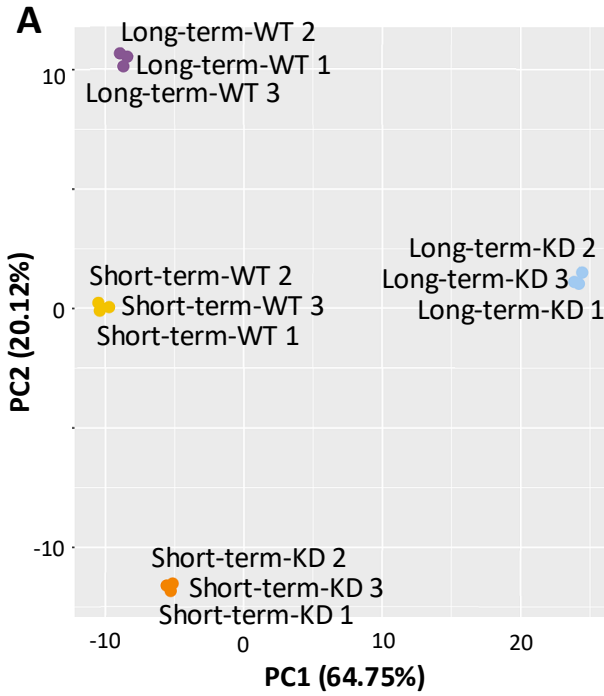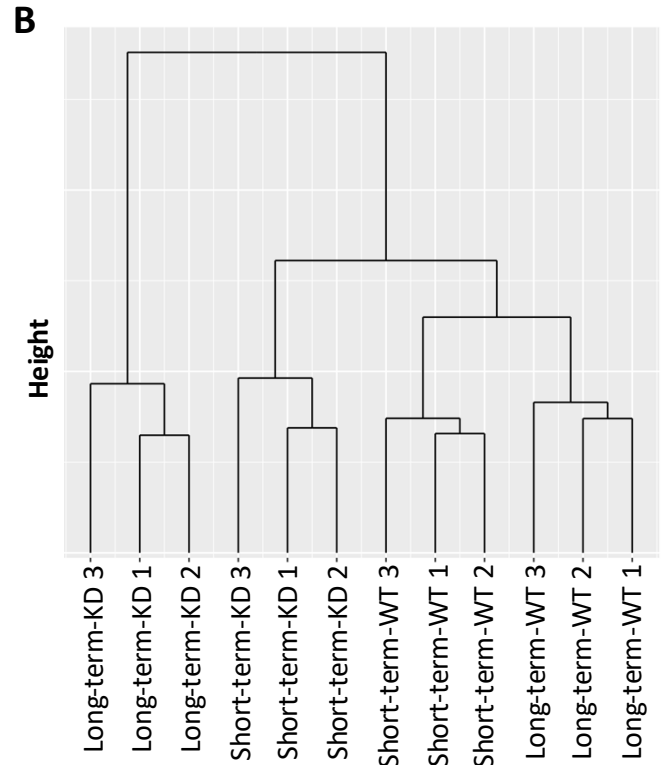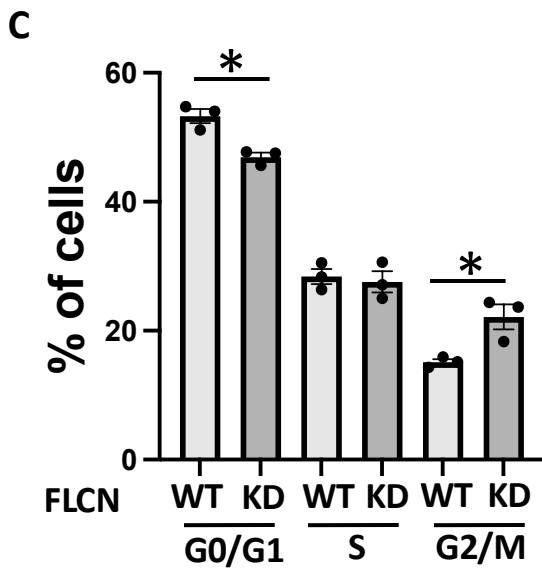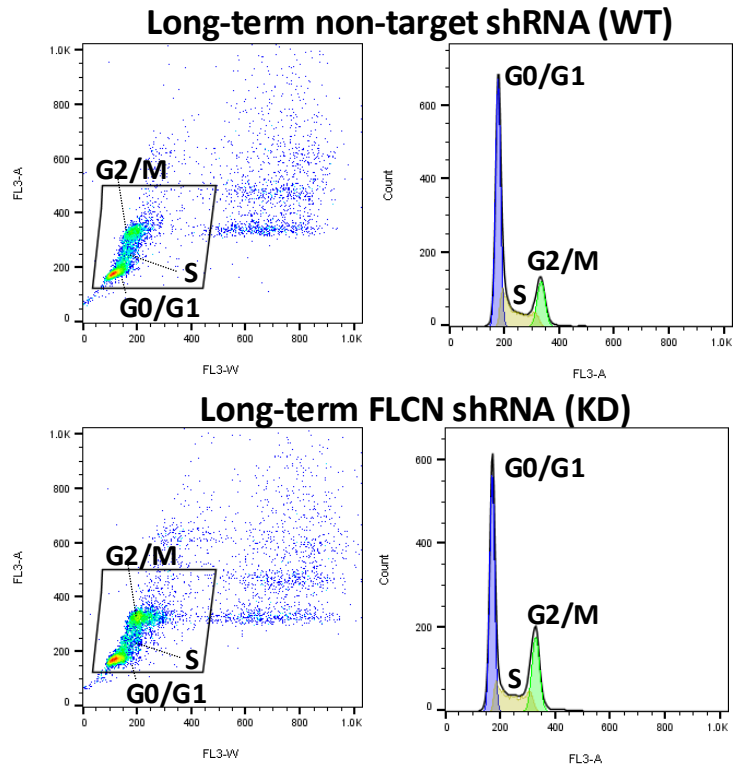

Supplement: Supplementary file 3 — Supplementary Figure 2 [file 41388_2025_3325_MOESM3_ESM.pdf]

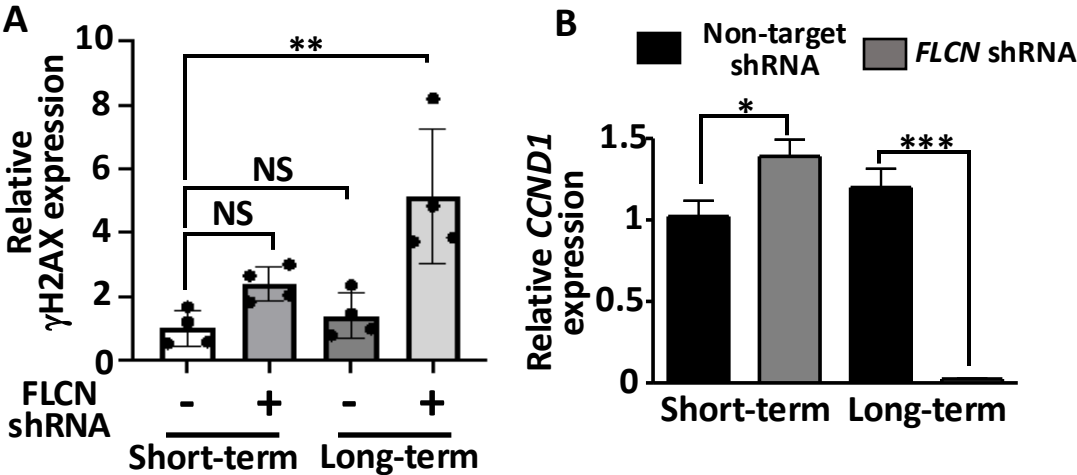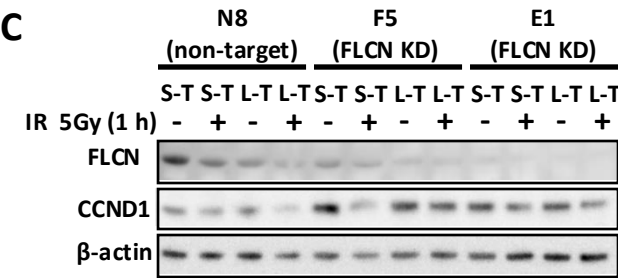

Supplement: Supplementary file 5 — Supplementary Figure 4 [file 41388_2025_3325_MOESM5_ESM.pdf]
