# Supplementary material for: Characterizing the tumor suppressor activity of FLCN in Birt-Hogg-Dubé syndrome cell models through transcriptomic and proteomic analysis
Source: Oncogene. 2025 Mar 25;44(23):1833–43. doi: 10.1038/s41388-025-03325-z (PMC12143978; doi:10.1038/s41388-025-03325-z)
Supplement: Supplementary file 4 — Supplementary Figure 3 [file 41388_2025_3325_MOESM4_ESM.pdf]

Supplementary Figure 3

A

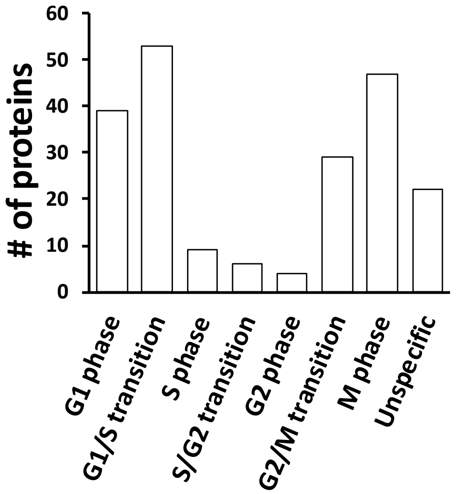

B

Protein Symbol	Protein Name	Unique peptides	Total peptides
**PRKDC**	Protein kinase DNA-activated, catalytic	66	147
**RIF1**	Rap1 interacting factor homology	7	11
**ATM**	ATM serine/threonine kinase	5	8
**ATR**	ATR serine/threonine kinase	2	3
**ASCC3**	Activating signal cointegrator 1 complex subunit 3	12	15
**DDB1**	Damage specific DNA binding protein 1	6	10
**RuvBL2**	RuvB like AAA ATPase 2	10	14
**RuvBL1**	RuvB like AAA ATPase 1	7	14
**P53**	Tumor protein p53	3	6
**TPP1**	Tripeptidyl peptidase I	2	4

C

>PRKDC

MAGSGAGVRCSLLRLQETLSAADRCGAALAGHQLIRGLGQECVLSSSPAVLALQTSLVFSRDFGLLVFVRKSLNSIEFRECREEILKFLCIFLEKMGQKIA
PYSVEIKNTCTSVYTKDRAAKCKIPALDLLIKLLQTFRSSRLMDEFKIGELFSKFYGELALKKKIPDTVLEKVYELLGLLGEVHPSEMINNAENLFRAFLG
ELKTQMTSAVREPKLPVLAGCLKGLSSLLCNFTKSMEEDPQTSREIFNFVLKAIRPQIDLKRYAVPSAGLRLFALHASQFSTCLLDNYVSLFEVLLKWCAH
TNVELKKAALSALESFLKQVSNMVAKNAEMHKNKLQYFMEQFYGIIRNVDSNNKELSIAIRGYGLFAGPCKVINAKDVDFMYVELIQRCKQMFLTQTDTGD
DRVYQMPSFLQSVASVLLYLDTVPEVYTPVLEHLVVMQIDSFPQYSPKMQLVCCRAIVKVFLALAAKGPVLRNCISTVVHQGLIRICSKPVVLPKGPESES
EDHRASGEVRTGKWKVPTYKDYVDLFRHLLSSDQMMDSILADEAFFSVNSSSESLNHLLYDEFVKSVLKIVEKLDLTLEIQTVGEQENGDEAPGVWMIPTS
DPAANLHPAKPKDFSAFINLVEFCREILPEKQAEFFEPWVYSFSYELILQSTRLPLISGFYKLLSITVRNAKKIKYFEGVSPKSLKHSPEDPEKYSCFALF
VKFGKEVAVKMKQYKDELLASCLTFLLSLPHNIIELDVRAYVPALQMAFKLGLSYTPLAEVGLNALEEWSIYIDRHVMQPYYKDILPCLDGYLKTSALSDE
TKNNWEVSALSRAAQKGFNKVVLKHLKKTKNLSSNEAISLEEIRIRVVQMLGSLGGQINKNLLTVTSSDEMMKSYVAWDREKRLSFAVPFREMKPVIFLDV
FLPRVTELALTASDRQTKVAACELLHSMVMFMLGKATQMPEGGQGAPPMYQLYKRTFPVLLRLACDVDQVTRQLYEPLVMQLIHWFTNNKKFESQDTVALL
EAILDGIVDPVDSTLRDFCGRCIREFLKWSIKQITPQQQEKSPVNTKSLFKRLYSLALHPNAFKRLGASLAFNNIYREFREEESLVEQFVFEALVIYMESL
ALAHADEKSLGTIQQCCDAIDHLCRIIEKKHVSLNKAKKRRLPRGFPPSASLCLLDLVKWLLAHCGRPQTECRHKSIELFYKFVPLLPGNRSPNLWLKDVL
KEEGVSFLINTFEGGGCGQPSGILAQPTLLYLRGPFSLQATLCWLDLLLAALECYNTFIGERTVGALQVLGTEAQSSLLKAVAFFLESIAMHDIIAAEKCF
GTGAAGNRTSPQEGERYNYSKCTVVVRIMEFTTTLLNTSPEGWKLLKKDLCNTHLMRVLVQTLCEPASIGFNIGDVQVMAHLPDVCVNLMKALKMSPYKDI
LETHLREKITAQSIEELCAVNLYGPDAQVDRSRLAAVVSACKQLHRAGLLHNILPSQSTDLHHSVGTELLSLVYKGIAPGDERQCLPSLDLSCKQLASGLL
ELAFAFGGLCERLVSLLLNPAVLSTASLGSSQGSVIHFSHGEYFYSLFSETINTELLKNLDLAVLELMQSSVDNTKMVSAVLNGMLDQSFRERANQKHQGL
KLATTILQHWKKCDSWWAKDSPLETKMAVLALLAKILQIDSSVSFNTSHGSFPEVFTTYISLLADTKLDLHLKGQAVTLLPFFTSLTGGSLEELRRVLEQL
IVAHFPMQSREFPPGTPRFNNYVDCMKKFLDALELSQSPMLLELMTEVLCREQQHVMEELFQSSFRRIARRGSCVTQVGLLESVYEMFRKDDPRLSFTRQS
FVDRSLLTLLWHCSLDALREFFSTIVVDAIDVLKSRFTKLNESTFDTQITKKMGYYKILDVMYSRLPKDDVHAKESKINQVFHGSCITEGNELTKTLIKLC
YDAFTENMAGENQLLERRRLYHCAAYNCAISVICCVFNELKFYQGFLFSEKPEKNLLIFENLIDLKRRYNFPVEVEVPMERKKKYIEIRKEAREAANGDSD
GPSYMSSLSYLADSTLSEEMSQFDFSTGVQSYSYSSQDPRPATGRFRRREQRDPTVHDDVLELEMDELNRHECMAPLTALVKHMHRSLGPPQGEEDSVPRD
LPSWMKFLHGKLGNPIVPLNIRLFLAKLVINTEEVFRPYAKHWLSPLLQLAASENNGGEGIHYMVVEIVATILSWTGLATPTGVPKDEVLANRLLNFLMKH
VFHPKRAVFRHNLEIIKTLVECWKDCLSIPYRLIFEKFSGKDPNSKDNSVGIQLLGIVMANDLPPYDPQCGIQSSEYFQALVNNMSFVRYKEVYAAAAEVL
GLILRYVMERKNILEESLCELVAKQLKQHQNTMEDKFIVCLNKVTKSFPPLADRFMNAVFFLLPKFHGVLKTLCLEVVLCRVEGMTELYFQLKSKDFVQVM
RHRDDERQKVCLDIIYKMMPKLKPVELRELLNPVVEFVSHPSTTCREQMYNILMWIHDNYRDPESETDNDSQEIFKLAKDVLIQGLIDENPGLQLIIRNFW
SHETRLPSNTLDRLLALNSLYSPKIEVHFLSLATNFLLEMTSMSPDYPNPMFEHPLSECEFQEYTIDSDWRFRSTVLTPMFVETQASQGTLQTRTQEGSLS
ARWPVAGQIRATQQQHDFTLTQTADGRSSFDWLTGSSTDPLVDHTSPSSDSLLFAHKRSERLQRAPLKSVGPDFGKKRLGLPGDEVDNKVKGAAGRTDLLR
LRRRFMRDQEKLSLMYARKGVAEQKREKEIKSELKMKQDAQVVLYRSYRHGDLPDIQIKHSSLITPLQAVAQRDPIIAKQLFSSLFSGILKEMDKFKTLSE
KNNITQKLLQDFNRFLNTTFSFFPPFVSCIQDISCQHAALLSLDPAAVSAGCLASLQQPVGIRLLEEALLRLLPAELPAKRVRGKARLPPDVLRWVELAKL
YRSIGEYDVLRGIFTSEIGTKQITQSALLAEARSDYSEAAKQYDEALNKQDWVDGEPTEAEKDFWELASLDCYNHLAEWKSLEYCSTASIDSENPPDLNKI
WSEPFYQETYLPYMIRSKLKLLLQGEADQSLLTFIDKAMHGELQKAILELHYSQELSLLYLLQDDVDRAKYYIQNGIQSFMQNYSSIDVLLHQSRLTKLQS
VQALTEIQEFISFISKQGNLSSQVPLKRLLNTWTNRYPDAKMDPMNIWDDIITNRCFFLSKIEEKLTPLPEDNSMNVDQDGDPSDRMEVQEQEEDISSLIR
SCKFSMKMKMIDSARKQNNFSLAMKLLKELHKESKTRDDWLVSWVQSYCRLSHCRSRSQGCSEQVLTVLKTVSLLDENNVSSYLSKNILAFRDQNILLGTT
YRIIANALSSEPACLAEIEEDKARRILELSGSSSEDSEKVIAGLYQRAFQHLSEAVQAAEEEAQPPSWSCGPAAGVIDAYMTLADFCDQQLRKEEENASVI
DSAELQAYPALVVEKMLKALKLNSNEARLKFPRLLQIIERYPEETLSLMTKEISSVPCWQFISWISHMVALLDKDQAVAVQHSVEEITDNYPQAIVYPFII
SSESYSFKDTSTGHKNKEFVARIKSKLDQGGVIQDFINALDQLSNPELLFKDWSNDVRAELAKTPVNKKNIEKMYERMYAALGDPKAPGLGAFRRKFIQTF
GKEFDKHFGKGGSKLLRMKLSDFNDITNMLLLKMNKDSKPPGNLKECSPWMSDFKVEFLRNELEIPGQYDGRGKPLPEYHVRIAGFDERVTVMASLRRPKR
IIIRGHDEREHPFLVKGGEDLRQDQRVEQLFQVMNGILAQDSACSQRALQLRTYSVVPMTSRLGLIEWLENTVTLKDLLLNTMSQEEKAAYLSDPRAPPCE
YKDWLTKMSGKHDVGAYMLMYKGANRTETVTSFRKRESKVPADLLKRAFVRMSTSPEAFLALRSHFASSHALICISHWILGIGDRHLNNFMVAMETGGVIG
IDFGHAFGSATQFLPVPELMPFRLTRQFINLMLPMKETGLMYSIMVHALRAFRSDPGLLTNTMDVFVKEPSFDWKNFEQKMLKKGGSWIQEINVAEKNWYP
RQKICYAKRKLAGANPAVITCDELLLGHEKAPAFRDYVAVARGSKDHNIRAQEPESGLSEETQVKCLMDQATDPNILGRTWEGWEPWM
