# Supplementary material for: Characterizing the tumor suppressor activity of FLCN in Birt-Hogg-Dubé syndrome cell models through transcriptomic and proteomic analysis
Source: Oncogene. 2025 Mar 25;44(23):1833–43. doi: 10.1038/s41388-025-03325-z (PMC12143978; doi:10.1038/s41388-025-03325-z)

Figure 3C

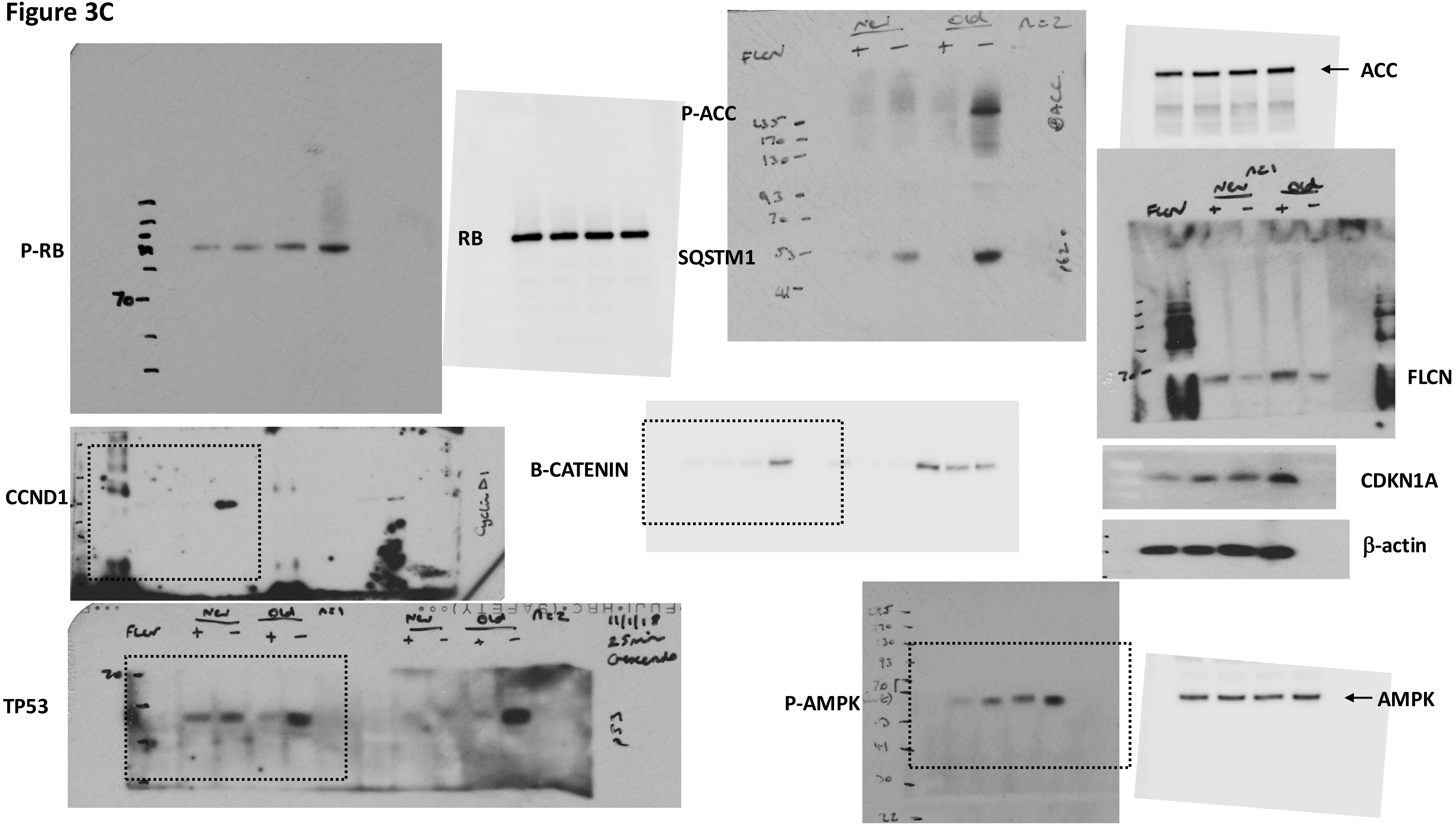
P-RB
RB
P-ACC
SQSTM1
ACC
FLCN
CCND1
B-CATENIN
CDKN1A
β-actin
TP53
P-AMPK
AMPK

Figure 5A

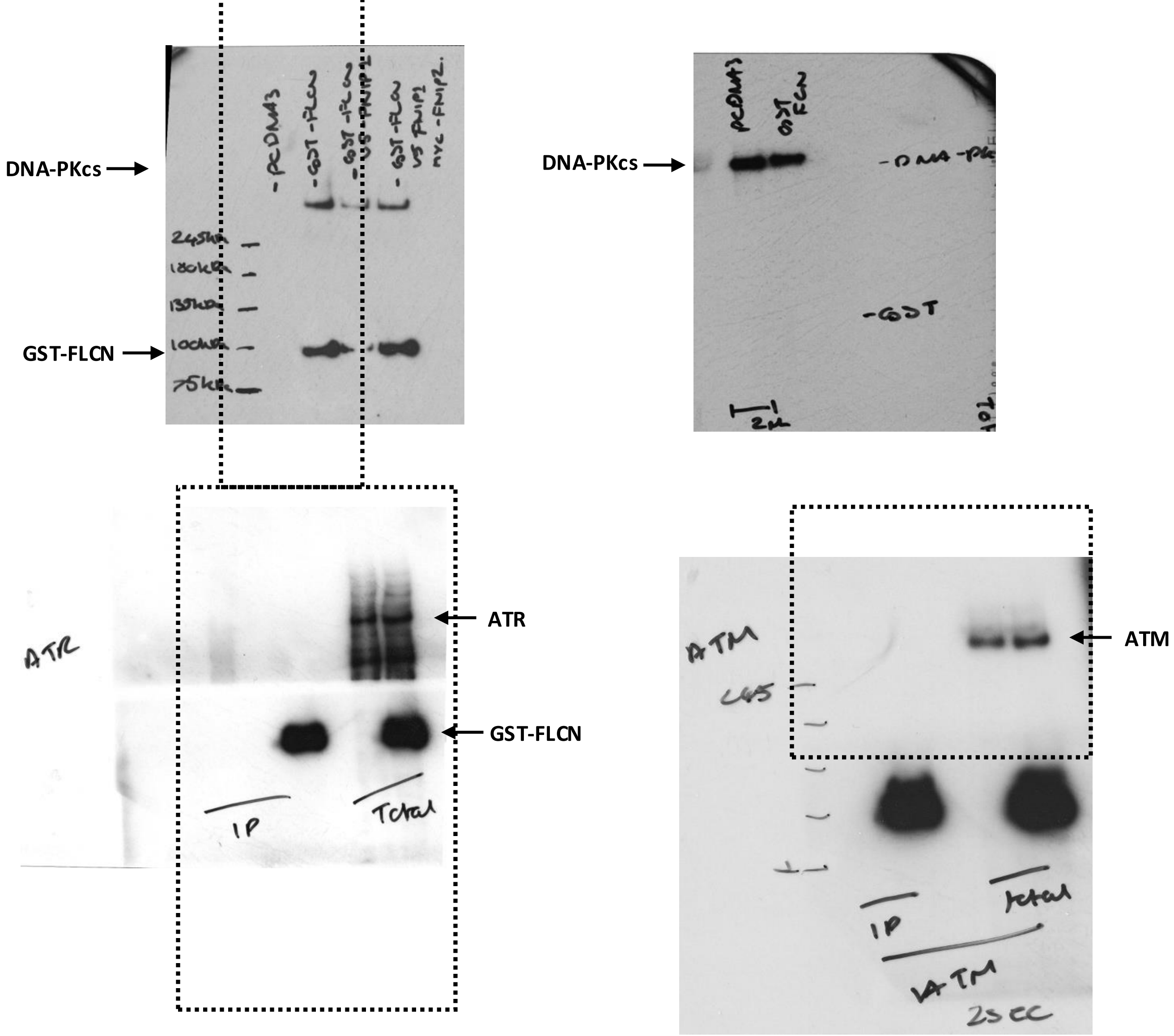

Figure 5B

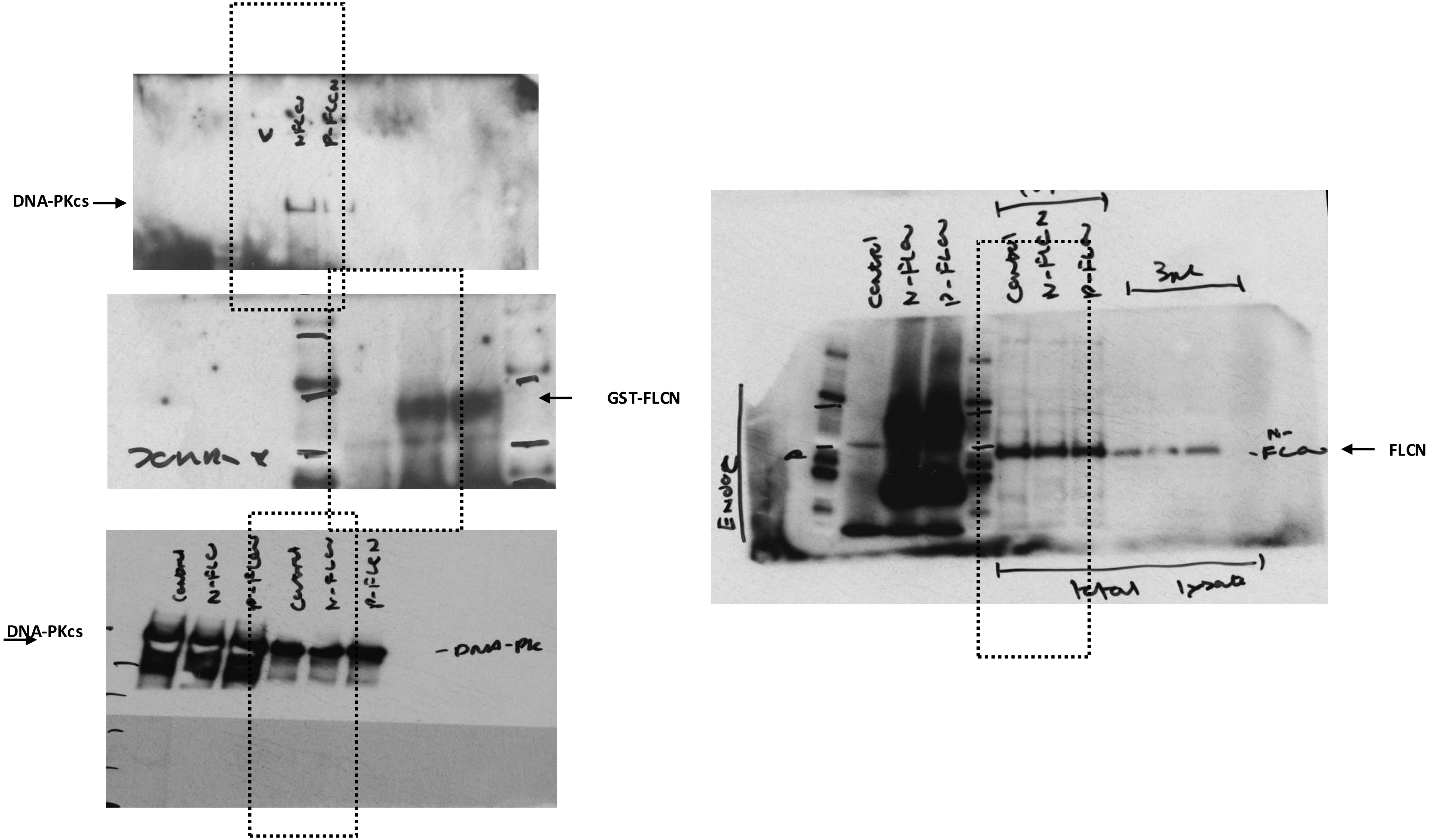

Figure 5C

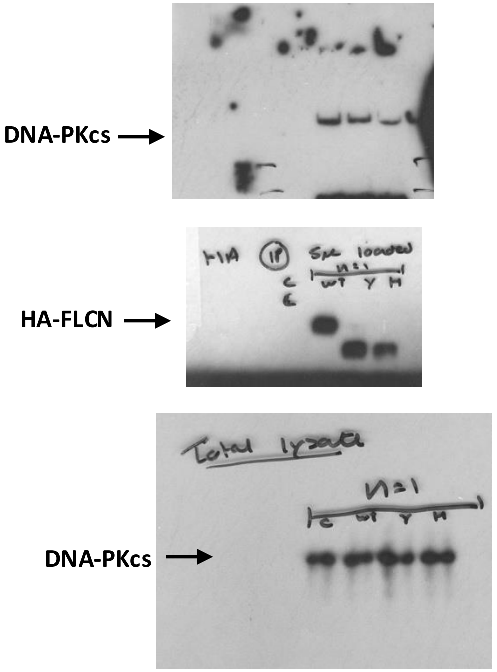

Figure 5D

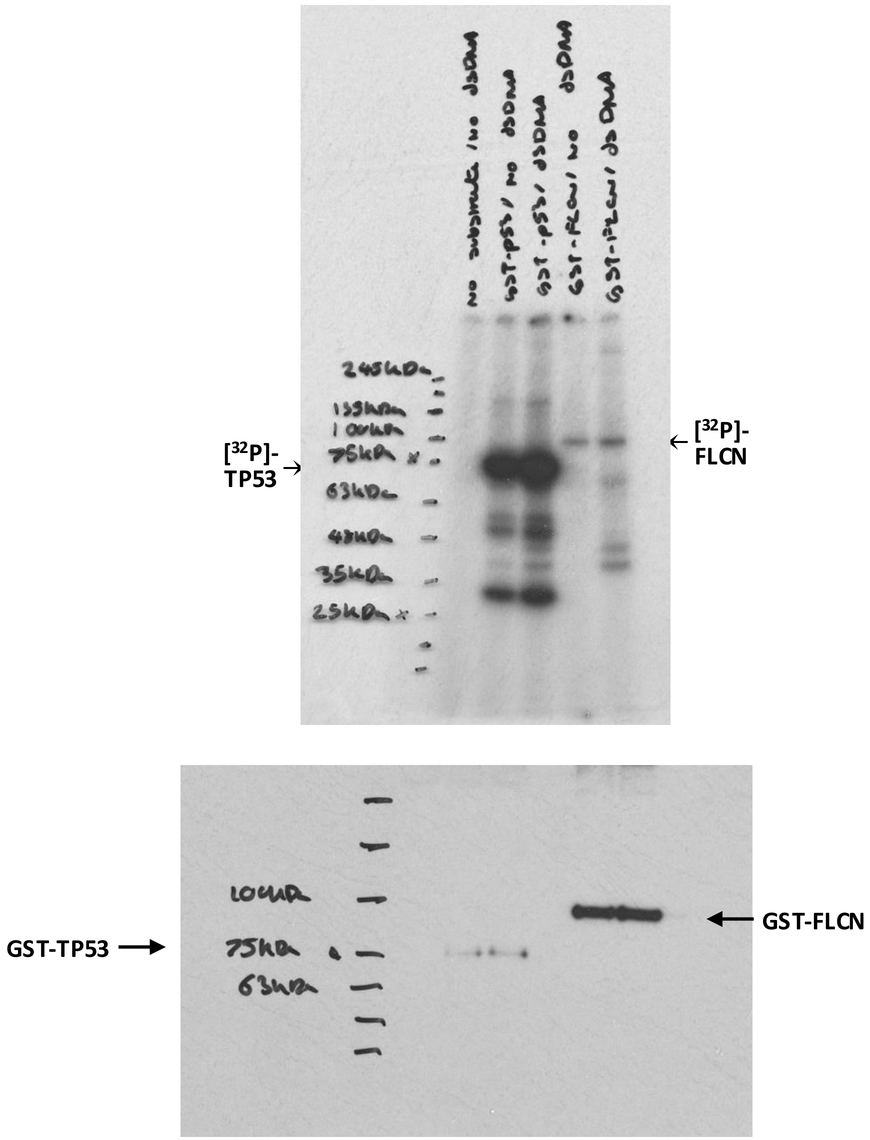

[32P]-TP53
[32P]-FLCN
GST-TP53
GST-FLCN
245kDa
135kDa
100kDa
75kDa
63kDa
48kDa
35kDa
25kDa

Figure 6A

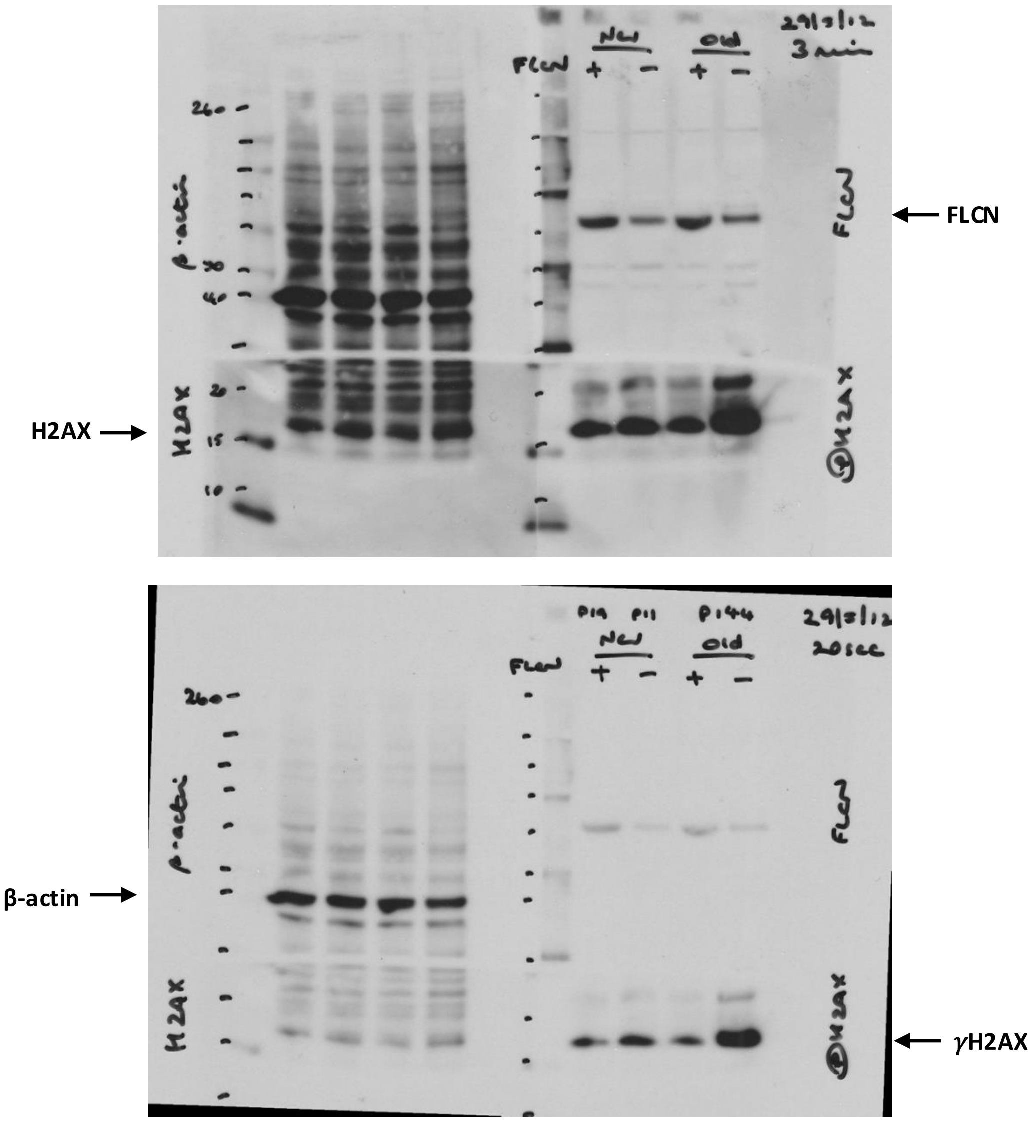

Figure 6B.

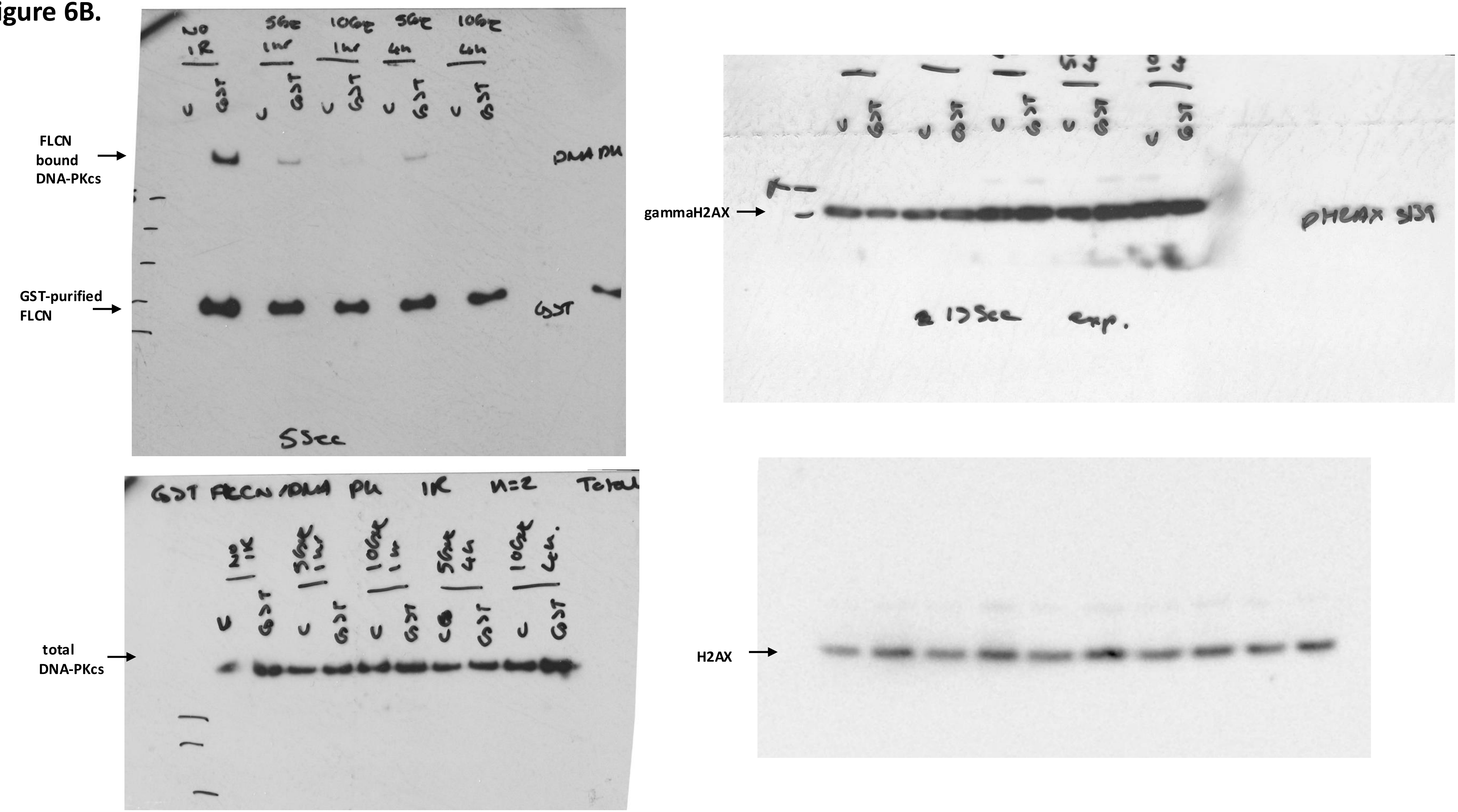
FLCN bound DNA-PKcs
GST-purified FLCN
gammaH2AX
total DNA-PKcs
H2AX

Figure 6C

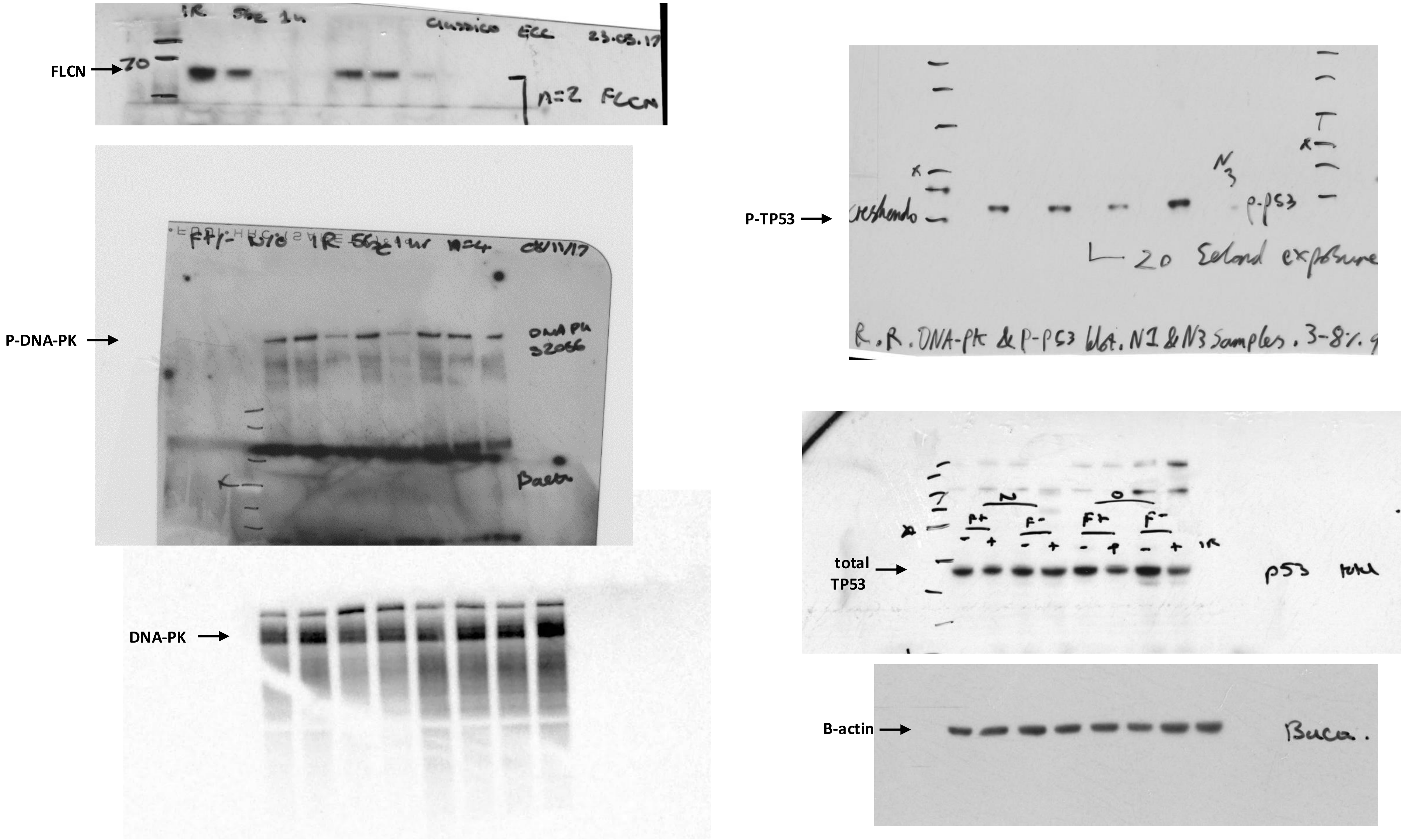

Figure 6C

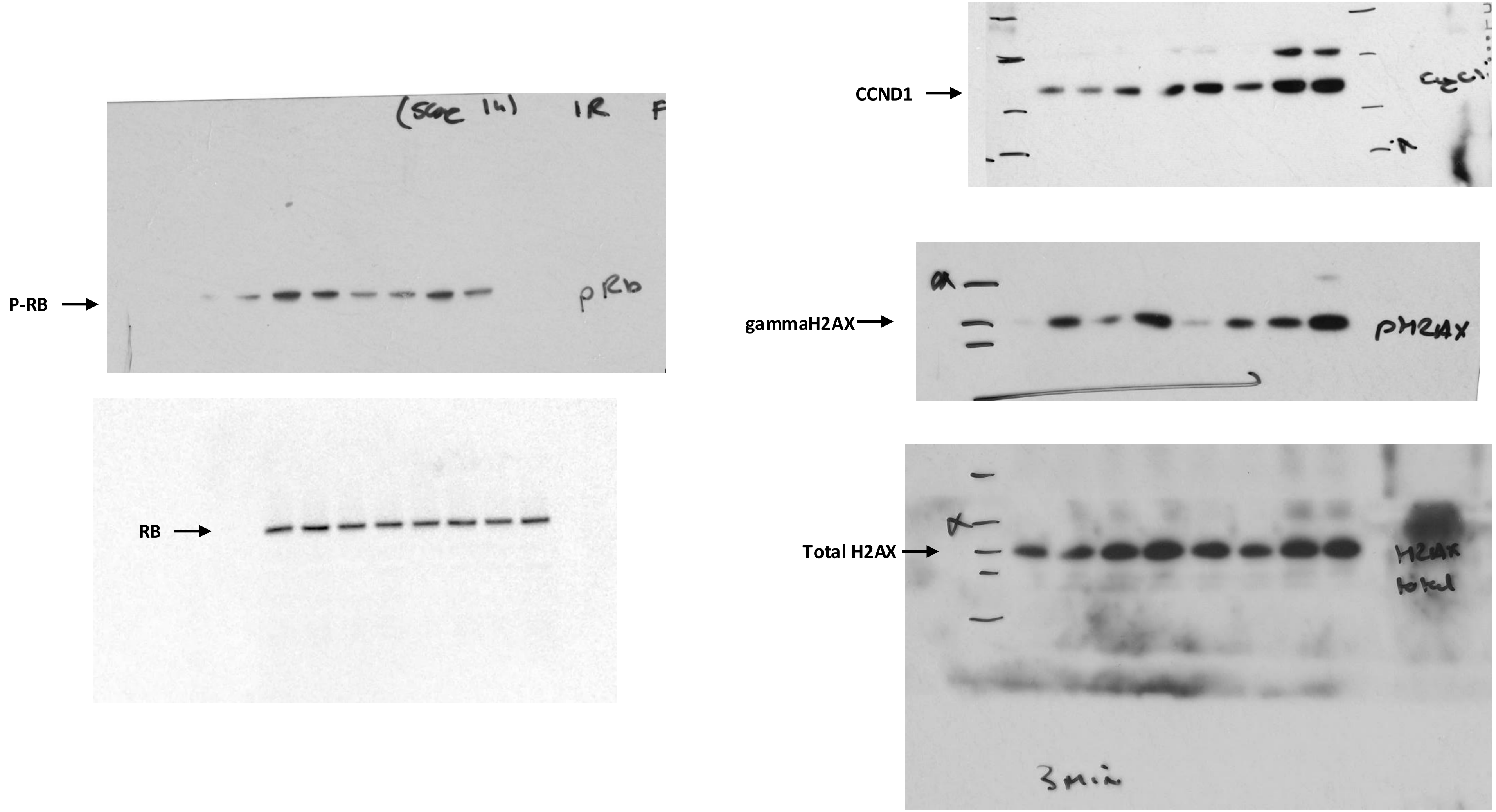

Supplementary Figure 4A: Blots for densitometry analysis (used non IR-treated sample in n2 and n3)

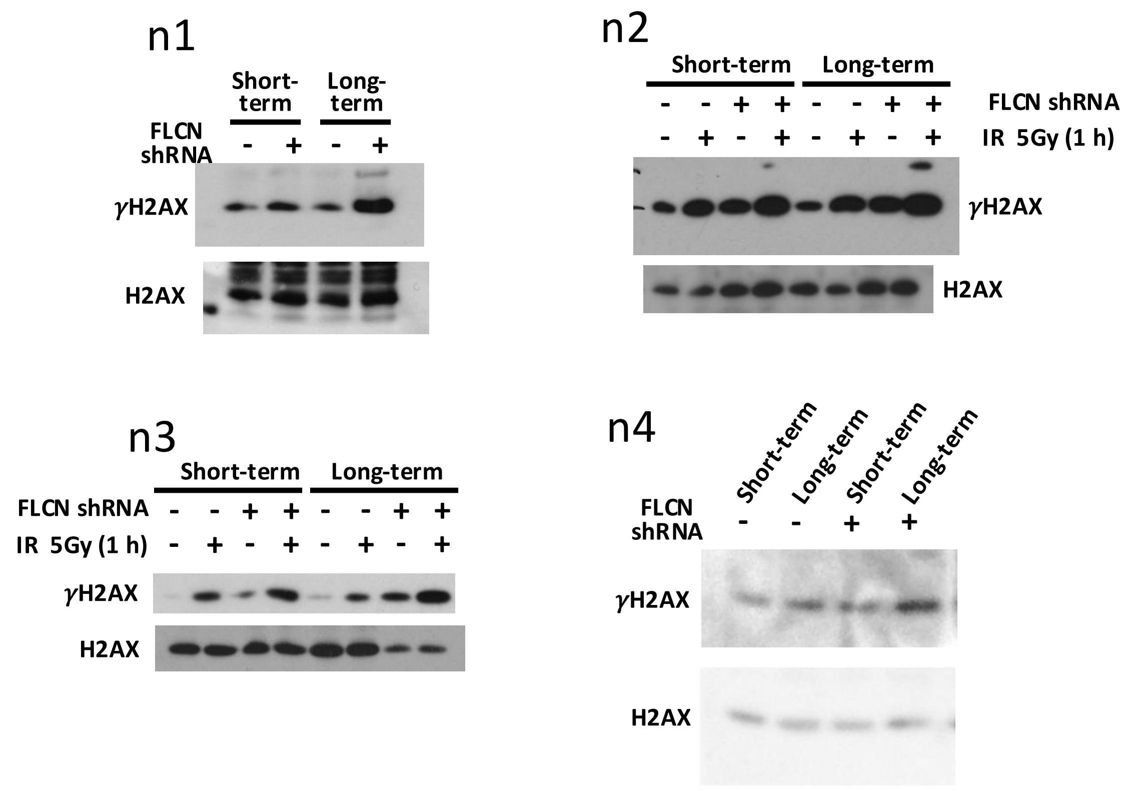

Supplementary Figure 4C

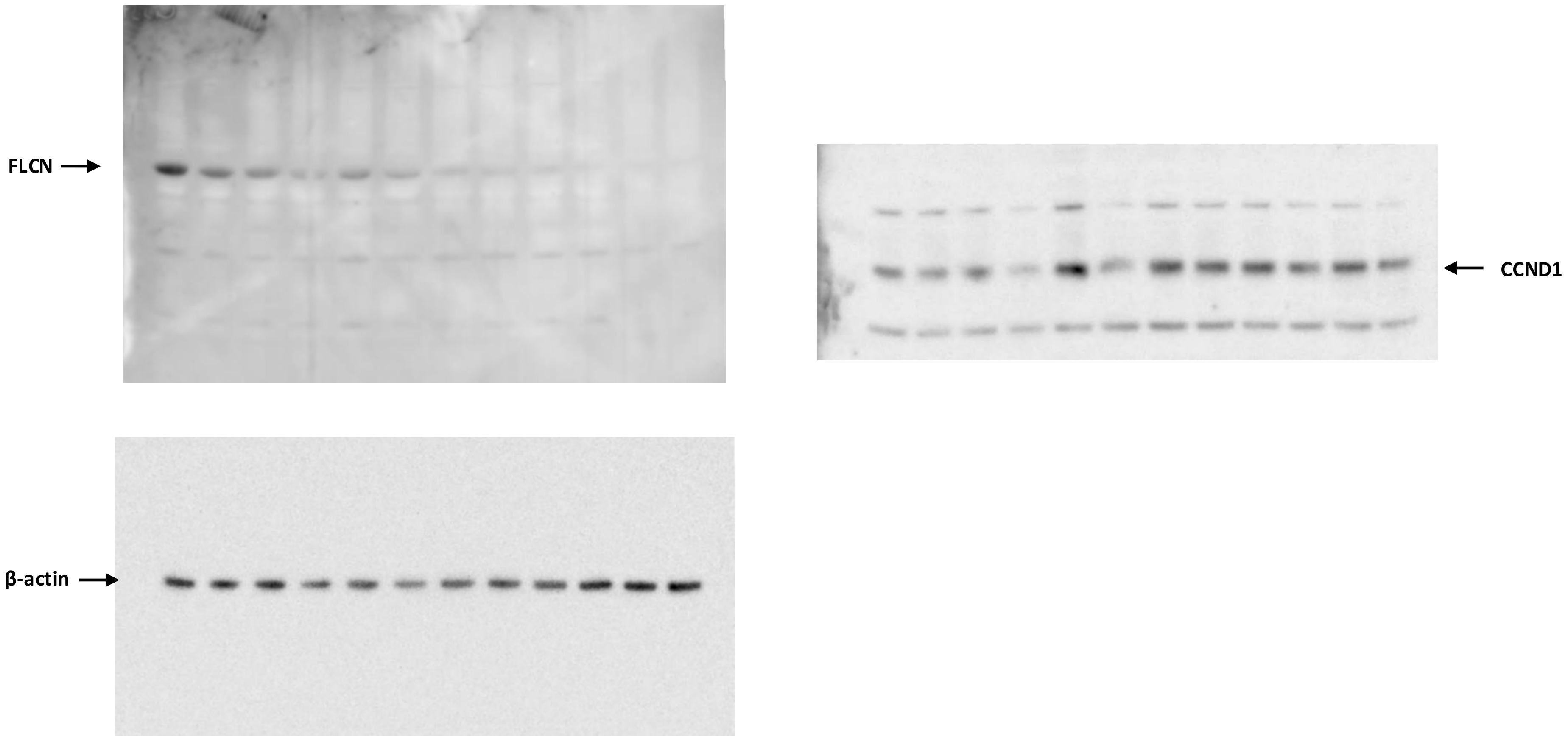

Supplement: Supplementary file 6 — Uncropped western blots [file 41388_2025_3325_MOESM6_ESM.pdf]
